# The incidence, risk factors, and prognosis of acute kidney injury in patients after cardiac surgery

**DOI:** 10.3389/fcvm.2024.1396889

**Published:** 2024-07-16

**Authors:** Xian-dong Wang, Rui Bao, Yang Lan, Zhen-zhen Zhao, Xin-yue Yang, Yun-yun Wang, Zhi-yong Quan, Jia-feng Wang, Jin-jun Bian

**Affiliations:** Faculty of Anesthesiology, Changhai Hospital, Naval Medical University, Shanghai, China

**Keywords:** acute kidney injury, cardiac surgery, risk factors, mean platelet volume, perioperative care

## Abstract

**Background:**

Acute kidney injury (AKI) represents a significant complication following cardiac surgery, associated with increased morbidity and mortality rates. Despite its clinical importance, there is a lack of universally applicable and reliable methods for the early identification and diagnosis of AKI. This study aimed to examine the incidence of AKI after cardiac surgery, identify associated risk factors, and evaluate the prognosis of patients with AKI.

**Method:**

This retrospective study included adult patients who underwent cardiac surgery at Changhai Hospital between January 7, 2021, and December 31, 2021. AKI was defined according to the Kidney Disease: Improving Global Outcomes (KDIGO) criteria. Perioperative data were retrospectively obtained from electronic health records. Logistic regression analyses were used to identify independent risk factors for AKI. The 30-day survival was assessed using the Kaplan–Meier method, and differences between survival curves for different AKI severity levels were compared using the log-rank test.

**Results:**

Postoperative AKI occurred in 257 patients (29.6%), categorized as stage 1 (179 patients, 20.6%), stage 2 (39 patients, 4.5%), and stage 3 (39 patients, 4.5%). The key independent risk factors for AKI included increased mean platelet volume (MPV) and the volume of intraoperative cryoprecipitate transfusions. The 30-day mortality rate was 3.2%. Kaplan–Meier analysis showed a lower survival rate in the AKI group (89.1%) compared to the non-AKI group (100%, *P* < 0.001).

**Conclusion:**

AKI was notably prevalent following cardiac surgery in this study, significantly impacting survival rates. Notably, MPV and administration of cryoprecipitate may have new considerable predictive significance. Proactive identification and management of high-risk individuals are essential for reducing postoperative complications and mortality.

## 1 Introduction

Acute kidney injury (AKI) is a significant complication following cardiac surgery, with reported incidence rates ranging from 5% to 42%, depending on various factors such as baseline characteristics, surgical types, and definitions employed (1). Moreover, postoperative dialysis may be required by up to 5.1% of the overall population (2). The precise underlying mechanism of cardiac surgery-associated AKI (CS-AKI) remains incompletely understood and involves an intricate interplay of factors including renal ischemia-reperfusion injury, inflammation, oxidative stress, and nephrotoxins (3). CS-AKI is associated with unfavorable outcomes such as prolonged ICU stay, increased hospitalization duration, a heightened risk of developing chronic kidney disease (CKD), and elevated mortality rates (4). Given the short- and long-term impact of AKI, early identification of high-risk individuals is crucial for preventing complications and reducing mortality.

Early identification and diagnosis of AKI still remain challenging. Timely recognition and diagnosis of AKI are crucial, as delayed detection has been identified as an independent risk factor for in-hospital mortality (5). Currently, the kidney disease improving global outcomes (KDIGO) criteria represent the prevailing epidemiological and clinical standard for diagnosing acute kidney injury, including CS-AKI (3). Serum creatinine, a significant clinical indicator of renal function and essential component of the KDIGO criteria, however, is recognized as an unreliable marker for early AKI detection, particularly during the initial stages when glomerular filtration rate alterations may not manifest immediately. This lag period required to achieve a steady state can hinder the accuracy of serum creatinine as an indicator of AKI onset. Another challenge arises from the potential dilution effect of intravenous fluid administration during the intraoperative period, which can further delay the diagnosis of AKI (6). All these factors diminish the predictive value of creatinine.

Recent studies have investigated several novel AKI biomarkers in patients undergoing cardiac surgery, providing new methods for the early diagnosis of AKI (7–11). Tissue inhibitor metalloproteinase-2·insulin-like growth factor-binding protein 7 (TIMP-2·IGFBP7) are markers of renal tubular stress, potentially detectable before the onset of tubular damage. It has been reported that the postoperative use of TIMP-2·IGFBP7 enhanced the prediction accuracy of CSA-AKI and could assist in identifying patients at risk of short-term adverse outcomes (8). Another study reported that urinary C-C motif chemokine ligand 14 (CCL14), a newly discovered biomarker for persistent acute kidney injury (AKI), may help improve the clinical management of AKI patients (9). However, the validity and broader applicability of these biomarkers require further clarification. At the same time, the costs associated with these tests also limit their widespread implementation in clinical practice.

Therefore, identifying new clinical markers as risk factors for AKI remains of paramount importance. Yet, recent clinical studies have mainly emphasized preoperative risk factors, incorporating only a limited number of intraoperative variables (2, 12–14). The multitude of potential covariates encountered during surgery might influence the incidence of AKI, thereby diminishing the efficacy of preoperative predictive indicators in diverse clinical scenarios.

The objective of this study was to determine the incidence of CS-AKI as defined by the KDIGO criteria in a tertiary hospital, and to analyze the perioperative risk factors associated with AKI. Furthermore, we aimed to establish the mortality rates to gain insight into the prognosis of AKI patients after cardiac surgery.

## 2 Materials and methods

This retrospective, observational, single-center, case-control study was conducted at a tertiary hospital in Shanghai, China, in accordance with the Strengthening the Reporting of Observational Studies in Epidemiology (STROBE) guidelines. The study protocol was approved by the institutional review board at hospital. As this study involved minimal risk and had a retrospective design, the requirement for informed consent was waived. All procedures performed in this study were in accordance with the ethical standards outlined in the Declaration of Helsinki.

### 2.1 Study population

The study cohort included all adult patients (aged 18 years or older) who underwent cardiac surgery at a tertiary hospital, Shanghai, China, between January 7th, 2021 and December 31st, 2021. The exclusion criteria were as follows: (a) patients with preoperative renal dysfunction, defined as a serum creatinine level above 176 μmol/L or the need for renal replacement therapy (13); (b)patients with history of a kidney transplantation; (c) patients with unavailable, incomplete, or invalid demographic, baseline, and perioperative data; (d) patients whose renal artery was involved in aortic disease or surgery; and (e) patients who died during the operation, or whose legally authorized representative requested discharge against medical advice on the first postoperative day due to critical condition, were excluded from the analysis.

### 2.2 Data collection and definition of outcome

All data were retrospectively obtained from the electronic health records and extracted by expert medical researchers who were unaware of the study hypothesis. The collected data encompassed demographic information, American Society of Anesthesiologists (ASA) physical status, perioperative laboratory test results, comorbidities, intraoperative details, and other perioperative data.

The primary outcome of this study was the occurrence of postoperative AKI at any stage, which was defined and staged according to the KDIGO classification. Secondary outcomes included the severity of AKI, the need for continuous renal replacement therapy (CRRT), 30-day mortality and in-hospital mortality. Following the KDIGO clinical practice guideline (15) and considering the patient's specific clinical situation, CRRT was initiated after a comprehensive assessment of the patient’s risks and benefits. Serum creatinine concentration values were recorded both before and after surgery. Preoperative serum creatinine concentration was determined based on the measurement obtained from the closest metabolic panel blood draw prior to the surgery. Due to the lack of available data on postoperative urine volumes and the usage of diuretics, urine output was not considered in the analysis. AKI stage 1 was defined as a rise in serum creatinine levels ≥26.4 µmol/L within 48 h or an increase to 1.5–1.9 times of the baseline value within 7 days. AKI stage 2 was defined as an increase in serum creatinine levels to 2.0–2.9 times of the baseline value, while AKI stage 3 was defined as an increase in serum creatinine levels to ≥3 times of the baseline value, an absolute increase in serum creatinine levels of ≥354 µmol/L, or the initiation of renal replacement therapy (RRT). The estimated creatinine-based glomerular filtration rate (eGFR) was calculated using the Modification of Diet in Renal Disease equation.

### 2.3 Anesthesia and perfusion management

Induction of anesthesia commonly involved a combination of propofol or etomidate, benzodiazepines, muscle relaxants, and opioids. During the surgical procedure, sevoflurane inhalation, opioids, and cis-atracurium were administered to maintain anesthesia. The standard roller pump cardiopulmonary bypass (CPB) circuit was deployed. Throughout CPB, perfusion flow rates were consistently maintained between 2.2 and 2.8 L/min/m^2^, mean arterial pressure was kept within 50–80 mmHg, and mixed venous oxygen saturation was ensured to be 75% or higher. Additionally, the activated clotting time (ACT) was effectively managed to surpass 480 s by using heparin. In most surgical procedures, the target for nasopharyngeal temperature was set between 34.5–35 °C and for bladder temperature between 34.5–35.5 °C. Notably, in aortic operations employing deep hypothermic circulatory arrest (DHCA), the nasopharyngeal temperature goal was adjusted to a range of 22 °C–25 °C, depending on the complexity and urgency of the surgery. Moreover, myocardial protection is achieved through the administration of cold blood cardioplegia.

### 2.4 Statistical analysis

Continuous variables were reported as mean ± standard deviation (SD) or median [interquartile range (IQR)], following the Shapiro-Wilk test to assess normal distribution. Categorical variables were presented as frequencies and proportions. To compare continuous variables, the Student *t*-test and Wilcoxon Rank Sum test were employed. For categorical variables, the Wilcoxon Rank Sum test, chi-square test, or Fisher's exact test were utilized. Differences between AKI-stage 1, 2, and 3 groups were assessed using either one-way ANOVA or Kruskal–Wallis test.

Nonlinear associations between continuous factors and postoperative AKI were evaluated using restricted cubic spline (RCS) models with four knots to flexibly model and visualize the relationships. For those factors showing nonlinearity, they were transformed into categorical variables based on the RCS models and commonly used clinical cut-off values. Three clinical factors with nonlinear associations were identified and are presented in Supplementary Figure S1. To assess multicollinearity among the variables, correlation coefficients and variance inflation factor were calculated. Logistic regression analyses were performed to identify independent risk factors for AKI. Initially, univariate analysis was conducted to select variables that were statistically significant for subsequent stepwise multivariate logistic regression analyses. *P* < 0.01 was considered as statistically signiﬁcant. The fit of the model was assessed by the Hosmer–Lemeshow good-of-fit test. The c-index, which equals the area under the receiver operating characteristics curve (AUC), was used to evaluate the discrimination of the model.

The unadjusted prognostic significance of AKI classification on event-free survival was assessed using the Kaplan-Meier method. Differences between survival curves were compared using the log-rank test. In a sensitivity analysis, cases undergoing off-pump cardiac surgery and heart transplantation were excluded, and the results remained consistent (see Supplementary Table). Statistical analyses were conducted using SPSS software version 21.0 (SPSS, Chicago, IL, USA) and R version 4.0.3 (R Foundation for Statistical Computing, Vienna, Austria).

## 3 Results

### 3.1 Characteristics of study cohorts

**Figure 1:**
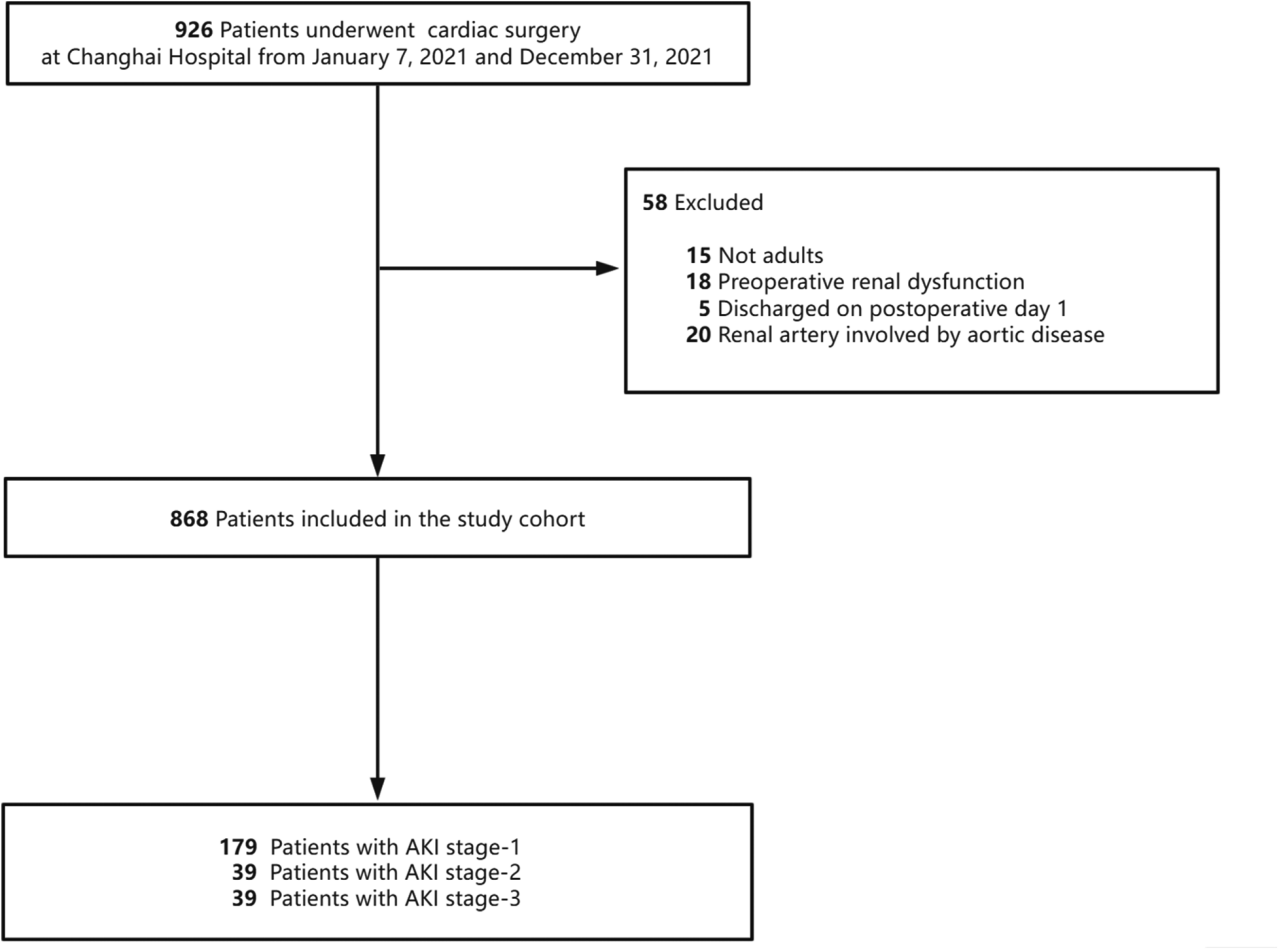
Study cohort used to explore the risk factors for acute kidney injury (AKI) after cardiac surgery.

The study included a total of 868 participants. The patient selection process for the study cohorts is illustrated in Figure 1. The mean age of the participants was 58.0 (50.0, 67.0) years, with 63.5% being male. Among the participants, 72.7% were classified as ASA ≥ 3. Table 1 provides a summary of the baseline characteristics, comorbidities, intraoperative factors, postoperative outcomes, and laboratory data of the participants. The baseline serum creatinine level was 73.00 (61.00, 85.00) µmol/L. Furthermore, the duration of surgery was recorded as 235.0 (200.0, 285.0) min. A total of 49 (5.6%) patients underwent off-pump cardiac surgery. Supplementary Table S1 provides the additional baseline data of the study population.

**Table 1:** Demographic data of the total population, patients with or without AKI.

	Total (*n* = 868)	Non-AKI (*n* = 611)	AKI (*n* = 257)	*p value*
Age (year), Median (IQR)	58.0 (50.0, 67.0)	57.0 (48.0, 66.0)	62.0 (53.0, 69.0)	< 0.001
Gender, *n* (%)				0.366
Male	551 (63.5)	382 (62.5)	169 (65.8)	
BMI (kg/m^2^), Median (IQR)	23.5 (21.5, 26.0)	23.5 (21.6, 25.8)	23.4 (21.4, 26.4)	0.784
ASA physical status, *n* (%)				< 0.001
ASA 1,2	237 (27.3)	208 (33.9)	29 (11.4)	
ASA ≥3	631 (72.7)	403 (66)	228 (88.7)	
Diabetes mellitus, *n* (%)	80 (9.2)	55 (9)	25 (9.8)	0.736
Hypertension *n* (%)	166 (19.1)	114 (18.6)	52 (20.4)	0.59
Chronic liver disease, *n* (%)	12 (1.4)	10 (1.6)	2 (0.8)	0.525
Atrial fibrillation, *n* (%)	119 (13.7)	62 (10.1)	57 (22.2)	<0.001
Preoperative ECMO/IABP/or both support, *n* (%)	9 (1.0)	3 (0.5)	6 (2.3)	0.023
EF (%), Median (IQR)	58.0 (49.8, 66.0)	59.0 (51.5, 66.0)	56.0 (44.0, 64.0)	<0.001
Cr (μmol/L), Median (IQR)	73.0 (61.0, 85.0)	71.0 (60.0, 82.0)	78.0 (65.0, 96.0)	<0.001
Estimated glomerular filtration rate [ml/(min*1.73 m^2^)], Median (IQR)	95.0 (78.2, 109.8)	97.6 (81.4, 111.4)	88.2 (70.0, 100.9)	<0.001
Albumin (g/L), Median (IQR)	41.0 (38.0, 44.0)	41.0 (39.0, 44.0)	40.0 (37.0, 43.0)	<0.001
Hemoglobin (g/L), Mean ± SD	133.9 ± 19.9	136.0 ± 18.3	128.9 ± 22.5	<0.001
MPV (fL), Mean ± SD	11.2 ± 1.3	11.1 ± 1.2	11.4 ± 1.4	0.002
Platelet count (10^9 ^/L), Median (IQR)	188.0 (148.0, 229.0)	194.0 (155.0, 233.0)	169.0 (132.0, 223.0)	<0.001
BNP (pg/ml), Median (IQR)	113.7 (48.8, 282.8)	86.8 (38.7, 227.0)	174.0 (88.9, 445.7)	<0.001
INR, Median (IQR)	1.0 (1.0, 1.1)	1.0 (1.0, 1.1)	1.1 (1.0, 1.2)	<0.001
Surgery-related characteristics				
Emergency operation, *n* (%)	78 (9.0)	29 (4.8)	49 (19.1)	<0.001
Off pump operation, *n* (%)	49 (5.6)	42 (6.9)	7 (2.7)	0.016
Surgical types, *n* (%)				<0.001
CABG only	183 (21.1)	150 (24.5)	33 (12.8)	
Single-valve replacement only	138 (15.9)	115 (18.8)	23 (8.9)	
Multiple-valve replacement surgery only	117 (13.5)	75 (12.3)	42 (16.3)	
Combined CABG-valve procedure	31 (3.6)	18 (2.9)	13 (5.1)	
Aortic procedure	122 (14.1)	65 (10.6)	57 (22.2)	
Heart transplantation	24 (2.8)	10 (1.6)	14 (5.4)	
Others	253 (29.1)	178 (29.1)	75 (29.2)	
Aortic dissection surgery, *n* (%)	49 (5.6)	14 (2.3)	35 (13.6)	<0.001
Intraoperative factors				
Intraoperative crystalloid infusion (ml), Median (IQR)	1,100.0 (1,100.0, 1,400.0)	1,100.0 (1,100.0, 1,300.0)	1,200.0 (1,100.0, 1,600.0)	<0.001
Intraoperative transfusion volume, Median (IQR)				
Total (unit)	0.0 (0.0, 6.0)	0.0 (0.0, 0.0)	3.0 (0.0, 20.0)	<0.001
Erythrocytes(ml)	0.0 (0.0, 0.0)	0.0 (0.0, 0.0)	0.0 (0.0, 400.0)	<0.001
Plasma(ml)	0.0 (0.0, 0.0)	0.0 (0.0, 0.0)	0.0 (0.0, 400.0)	<0.001
Platelet (unit)	0.0 (0.0, 0.0)	0.0 (0.0, 0.0)	0.0 (0.0, 10.0)	<0.001
Cryoprecipitate (unit)	0.0 (0.0, 0.0)	0.0 (0.0, 0.0)	0.0 (0.0, 10.0)	<0.001
Intraoperative blood loss(ml), Median (IQR)	300.0 (200.0, 500.0)	300.0 (200.0, 400.0)	300.0 (200.0, 500.0)	0.001
Intraoperative urine output(ml), Median (IQR)	1,000.0 (700.0, 1,500.0)	1,000.0 (700.0, 1,400.0)	1,000.0 (700.0, 1,600.0)	0.107
Duration of surgery(min), Median (IQR)	235.0 (200.0, 285.0)	225.0 (190.0, 265.0)	265.0 (225.0, 330.0)	<0.001
DHCA, *n* (%)	10 (1.2)	5 (0.8)	5 (2)	0.174
Minimum intraoperative Hb level (g/L), Median (IQR)	7.2 (6.1, 8.6)	7.4 (6.2, 8.8)	7.0 (5.9, 8.2)	0.005
Minimum intraoperative Hct level (%), Median (IQR)	23.0 (20.0, 26.0)	23.0 (20.0, 27.0)	22.0 (19.0, 25.0)	<0.001
Minimum intraoperative PaO_2_(mmHg), Median (IQR)	259.0 (166.0, 309.5)	260.0 (171.0, 312.0)	252.5 (147.0, 307.0)	0.131
Maximum intraoperative lactate level (mmol/L), Median (IQR)	2.7 (2.0, 3.9)	2.5 (1.9, 3.4)	3.5 (2.5, 5.6)	<0.001

BMI, body mass index; ASA, American Society of Anesthesiologists; ECMO, extra-corporeal membrane oxygenation; IABP, intra-aortic balloon pump; EF, ejection fraction; Cr, creatinine; MPV, mean platelet volume; INR, international normalized ratio; BNP, brain natriuretic peptide; CABG, coronary artery bypass graft surgery; DHCA, deep hypothermic circulatory arrest; Hb, hemoglobin; Hct, hematocrit; PaO_2_, partial pressure of oxygen in arterial blood; AKI, acute kidney injury.

### 3.2 Incidence and severity of AKI after cardiac surgery

A total of 257 patients (29.6%) in the study cohort developed stage 1 or worse AKI according to the KDIGO classification following the surgical procedure. The distribution of AKI stages among these patients was as follows: stage 1–179 patients (20.6%), stage 2–39 patients (4.5%), and stage 3–39 patients (4.5%). The rate of CRRT after surgery was 2.9% (25 patients). The AKI population was significantly younger than the non-AKI population, with a median age of 57.0 years compared to 62.0 years (*P* < 0.001). Patients with AKI were more likely to be classified as ASA III or greater (88.7% vs. 66.0%, *P* < 0.001) and were more frequently complicated with atrial fibrillation (22.3% vs. 10.1%, *P* < 0.001). Baseline laboratory and preoperative test results differed significantly between patients with and without AKI, as shown in Table 1. The preoperative serum creatinine levels were 78.0 (65.0–96.0) µmol/L in AKI patients, whereas they were only 71.0 (60.0–82.0) µmol/L in non-AKI patients. Baseline brain natriuretic peptide (BNP), international normalized ratio (INR), and hemoglobin (Hb) levels, analyzed as categorical variables based on RCS models (Supplementary Figure S1) and commonly used clinical cut-off values, also showed significant differences between the groups.

In the analysis of intraoperative factors, AKI patients received higher doses of blood products and underwent longer surgical procedures. Compared to non-AKI patients, AKI patients had significantly higher maximum intraoperative lactate levels (median of 3.5 vs. 2.5 mmol/L, *P* < 0.001), lower minimum intraoperative Hb levels (median of 7.0 vs. 7.4 g/dl, *P* < 0.001), and lower minimum intraoperative Hct levels (median of 22.0% vs. 23.0%, *P* < 0.001).

### 3.3 Outcome of AKI after cardiac surgery

**Figure 2:**
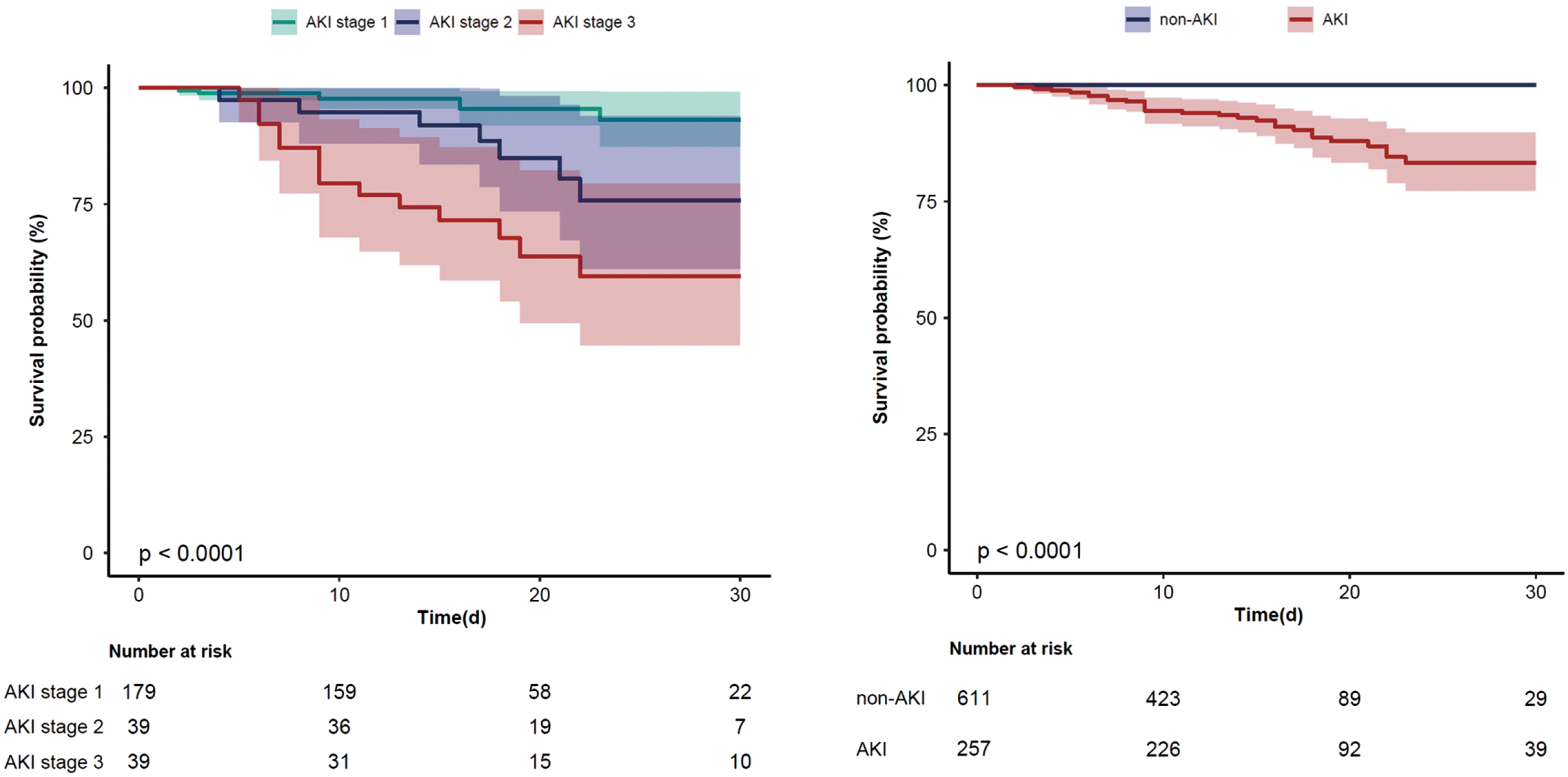
The Kaplan–Meier curve for 30-day mortality in all AKI stages patients and AKI/non-AKI patients (95% confidence interval).

Regarding postoperative outcomes, the AKI group displayed a significantly higher incidence of postoperative complications, including cardiac arrest (n% of 3.5 vs. 0.3, *P* < 0.001), reintubation (n% of 6.6 vs. 1.1, *P* < 0.001), tracheostomy (n% of 5.4 vs. 0, *P* < 0.001), 30-day mortality (n% of 10.9 vs. 0, *P* < 0.001) and in-hospital mortality (n% of 12.8 vs. 0.2, *P* < 0.001), relative to the non-AKI group. Figure 2 illustrates the comparative incidence of postoperative AKI and 30-day mortality. Notably, patients with the most severe AKI had the lowest event-free survival rates compared to those with AKI stage 1 or 2. Additional patient characteristics are presented in Table 2, Supplementary Tables S2, S3.

**Table 2:** Postoperative outcomes of the total population, patients with or without AKI.

	Total (*n* = 868)	Non-AKI (*n* = 611)	AKI (*n* = 257)	*p* value
Postoperative outcomes				
Duration of mechanical ventilation in ICU (h), Median (IQR)	8.5 (4.5, 20.0)	6.0 (4.3, 16.8)	19.0 (9.0, 24.1)	<0.001
>24 h	118 (13.6)	45 (7.4)	73 (28.4)	<0.001
>48 h	49 (5.6)	10 (1.6)	39 (15.2)	<0.001
Reintubation, *n* (%)	24 (2.8)	7 (1.1)	17 (6.6)	<0.001
Tracheostomy, *n* (%)	14 (1.6)	0 (0)	14 (5.4)	<0.001
Maximum postoperative PCT level (ng/ml), Median (IQR)	1.2 (0.5, 4.2)	0.9 (0.4, 1.9)	4.1 (1.1, 10.9)	<0.001
Initiation of CRRT, *n* (%)	25 (2.9)	0 (0)	25 (9.7)	<0.001
Cardiac arrest, *n* (%)	11 (1.3)	2 (0.3)	9 (3.5)	<0.001
Redo surgery, *n* (%)	47 (5.4)	23 (3.8)	24 (9.4)	<0.001
Postoperative ECMO/IABP/or both support, *n* (%)	53 (6.1)	12 (2)	41 (16)	<0.001
LOS-ICU(d), Median (IQR)	3.0 (2.0, 5.0)	2.0 (1.0, 3.0)	5.0 (3.0, 9.0)	<0.001
LOS (d), Median (IQR)	19.0 (15.0, 25.2)	17.0 (14.0, 23.0)	23.0 (18.5, 31.5)	<0.001
Postoperative LOS(d), Median (IQR)	13.0 (9.0, 18.0)	12.0 (9.0, 16.0)	16.0 (12.0, 23.0)	<0.001
In-hospital mortality, *n* (%)	34 (3.9)	1 (0.2)	33 (12.8)	<0.001
30-day mortality, *n* (%)	28 (3.2)	0 (0)	28 (10.9)	<0.001

ICU, intensive care unit; PCT, procalcitonin; CRRT, continuous renal replacement therapy; LOS, length of stay; LOS-ICU, length of stay in ICU; AKI, acute kidney injury.

### 3.4 Risk factors for AKI in patients after cardiac surgery

**Figure 3:**
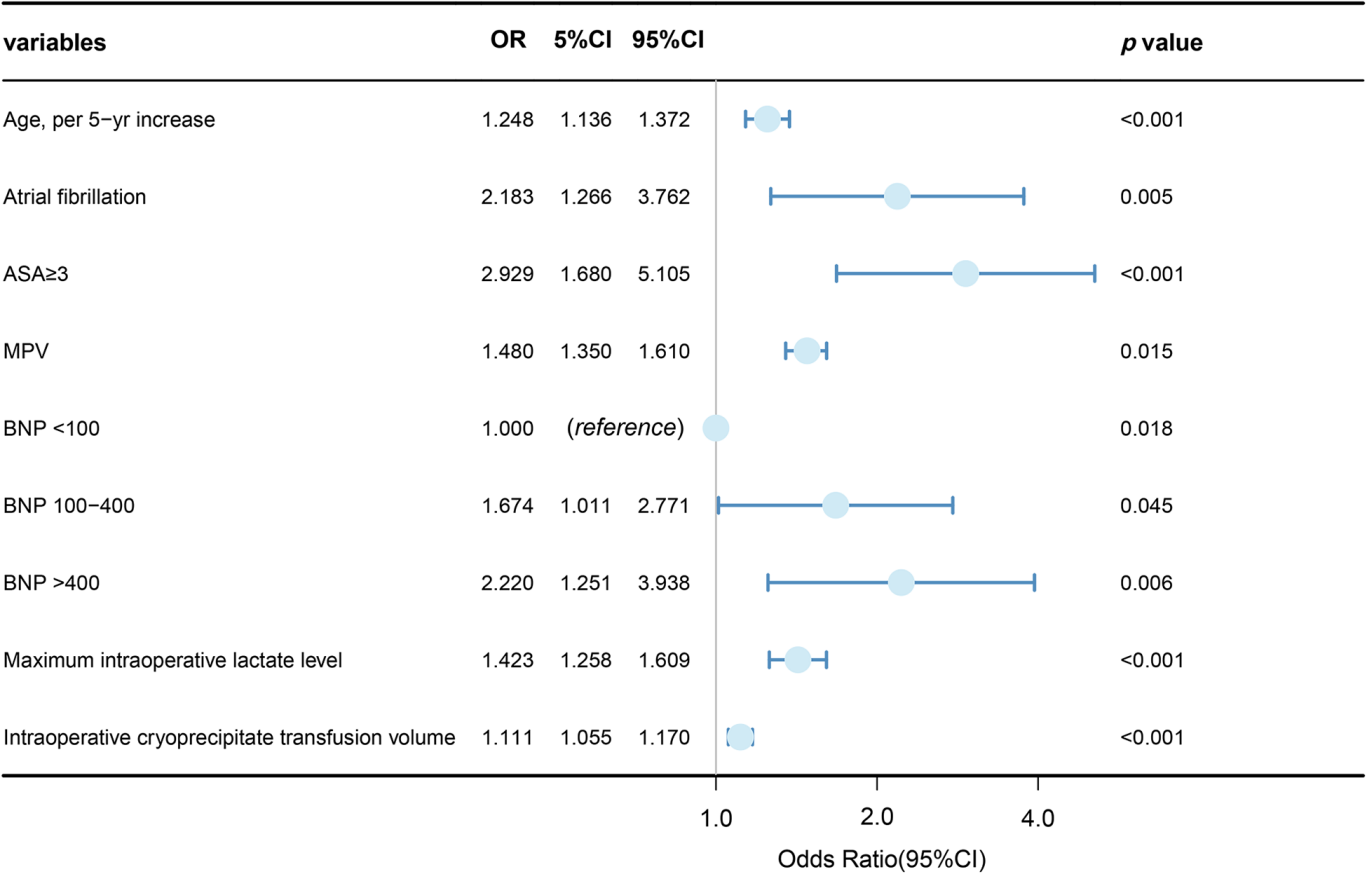
Logistic multivariable regression analysis showing the risk variables of AKI after cardiac surgery.

**Figure 4:**
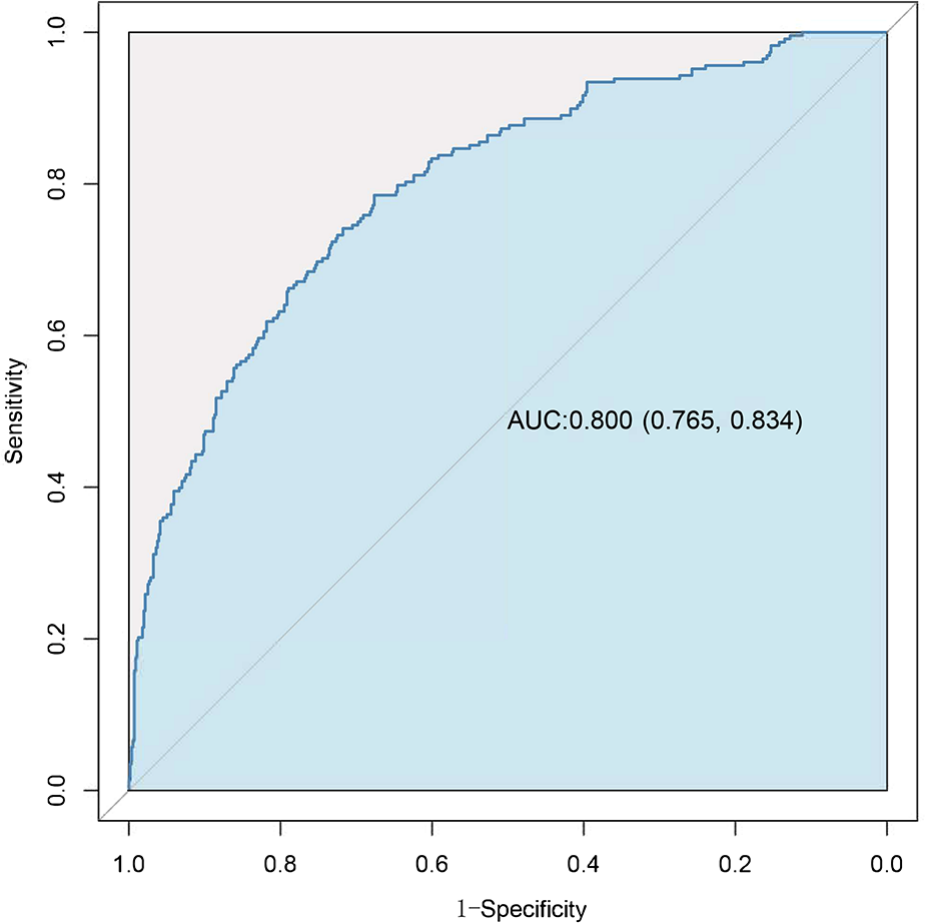
Receiver-operating characteristic (ROC) curve for evaluating the discrimination performance of the logistic model in the study cohort.

Variables showing a significant statistical difference (*P* < 0.01) in the univariate analyses were included in a stepwise forward logistic regression model, as shown in Supplementary Table S4. In the overall study cohort, multivariate logistic regression analysis identified several independent predictors for AKI following cardiac surgery. Significant predictors included maximum intraoperative lactate level [odds ratio (OR): 1.423, 95% CI: 1.258–1.609; *P* < 0.001], age (OR: 1.248, 95% CI: 1.136–1.372; *P* < 0.001), atrial fibrillation (OR: 2.183, 95% CI: 1.266–3.762; *P* = 0.05), intraoperative cryoprecipitate transfusion volume (OR: 1.111, 95% CI: 1.055–1.170; *P* < 0.001), ASA classification ≥3 (OR: 2.929, 95% CI: 1.680–5.105; *P* < 0.001), MPV (OR: 1.148, 95% CI: 1.350–1.610; *P* = 0.015), and higher BNP levels (BNP 100–400 vs. BNP < 100, OR: 1.674, 95% CI: 1.011–2.771; BNP >400 vs. BNP < 100, OR: 2.220, 95% CI: 1.251–3.938). Figure 3 showed the results of the multivariate analysis for AKI predictors. The logistic model exhibited robust discrimination, with an AUC of 0.800 (95% CI 0.765–0.834) (Figure 4). The Hosmer–Lemeshow goodness-of-fit test indicated good calibration of the logistic model (*P* = 0.961). In a sensitivity analysis, exclusion of patients undergoing heart transplants and off-pump surgeries from the study population yielded similar results (see Supplementary Tables S5–S10).

## 4 Discussion

This retrospective study presented an analysis of the incidence and associated risk factors of AKI following cardiac surgery. Through the utilization of a multivariate logistic regression analysis, our findings revealed the significant independent association of seven variables with CSA-AKI. These identified factors exhibited robust discrimination performance in predicting the occurrence of CSA-AKI, and MPV and administration of cryoprecipitate may have new considerable predictive significance. After sensitivity analyses, we derived similar conclusions. These risk factors are easily monitored and available, and of significant clinical value for guiding perioperative renal protection strategies. Moreover, our study further established a statistically significant relationship between the presence and severity of AKI and subsequent 30-day mortality following cardiac surgery. This study was conducted at a large tertiary hospital that specializes in complex and various types of cardiac surgeries, enhancing the credibility and generalizability of the study results.

Among the patients included in this study, it was observed that 257 individuals (29.6%) developed AKI, which was consistent with the incidence rates reported in other studies (1, 2, 16). This study demonstrated that the in-hospital mortality rate and 30-day mortality rate of patients with AKI were significantly higher than those of non-AKI individuals. Patients with AKI exhibited poorer clinical outcomes. Importantly, early identification and timely intervention in AKI, as well as allocating relatively more clinical care to AKI patients, may help improve their clinical outcomes.

Several major underlying injury pathways are involved in the development of CSA-AKI, including hypoperfusion, ischemia-reperfusion injury, neurohumoral activation, inflammation, oxidative stress, nephrotoxins, and mechanical factors (3). Recent studies have focused on investigating clinical risk factors associated with AKI following cardiac surgery. However, these studies have predominantly concentrated on preoperative risk factors and have limited inclusion of intraoperative variables (2, 12–14). In our study, we aimed to provide a more comprehensive evaluation by incorporating detailed intraoperative variables. Our findings revealed that out of the numerous variables examined, only seven remained statistically significant in the final multivariate logistic model. Notably, two of these significant predictors were intraoperative variables: maximum intraoperative lactate level and intraoperative cryoprecipitate transfusion volume. Some of these results are consistent with previous research, which has also identified older age and higher ASA scores as factors associated with AKI after cardiac surgery (2, 13, 14). Age has consistently emerged as a crucial risk factor for CSA-AKI, underscoring the importance of prioritizing post-operative cardiac kidney injury in elderly patients (17). Additionally, it is worth noting that patients aged over 70 years undergoing cardiac surgery have been found to exhibit three times higher odds of long-term mortality compared to their younger counterparts (1). These findings indicate that patients of advanced age and those with higher ASA scores are more prone to comorbidities and complications, warranting greater clinical attention.

The logistic regression analysis conducted in our study has identified that a higher intraoperative lactate level is a significant risk factor for CS-AKI. Elevated lactate levels are commonly regarded as an indicator of inadequate tissue perfusion. In a study by Juan et al. (17), involving the analysis of 2,940 patients, it was found that higher arterial lactate levels 24 h after admission were independently associated with postoperative AKI in cardiac surgery patients. Similarly, Hauer et al. (18) reported that a serum lactate level exceeding 1.1 mmol/L within the first 24 h following surgery proved to be the strongest predictor for the development of renal failure after cardiac surgery. Furthermore, various factors such as procedures involving CPB, hemodilution, hypothermia, low-flow CPB, and excessive neurohormonal activation have also been linked to the occurrence of lactic acidosis during CPB (19, 20). Hence, perioperative lactate levels should be given due attention as they represent a significant risk factor for the development of AKI. This easily available and measurable indicator has important clinical implications, emphasizing that ensuring adequate organ perfusion and oxygenation, along with maintaining homeostasis, is fundamental to protecting renal function. Preoperative optimization of hemoglobin levels, appropriate intraoperative CPB perfusion and ventilation strategies, monitoring of blood gas analysis, and maintaining homeostasis may be effective measures to reduce the risk of renal injury.

Platelets play a critical role in acute hemostasis and inflammation and are associated with various inflammatory diseases (21–23). They adhere to the endothelial wall, modify vascular permeability, recruit and interact with leukocytes, and activate the complement system, all of which significantly contribute to the hemodynamic and inflammatory processes of AKI (23). Jansen et al. (24) reported that platelet activation, platelet-neutrophil interaction, and neutrophil extracellular trap (NET) formation lead to renal inflammation and further kidney injury. MPV is a simple and cost-effective measure conducted by hematological analyzers. It is increasingly recognized as an important parameter of platelet function and activity. Platelets with elevated MPV are assumed to be younger and more reactive (25), containing more α-granules, including thrombospondin, P-selectin, and platelet factor 4, as well as several factors involved in coagulation. These prothrombotic substances can aggravate inflammation when released (23, 26). While several previous studies have reported that increased MPV is a significant prognostic risk factor in AKI and critically ill patients (27), few studies have investigated the association between MPV and the occurrence of AKI after cardiac surgery. The results of our study suggest that a higher baseline MPV (OR: 1.148, 95% CI: 1.350–1.610; *P* = 0.015) was associated with an increased risk of AKI. Abinaya et al. (28) retrospectively analyzed 4,204 patients who underwent cardiac surgery and found an independent association between the magnitude of postoperative MPV changes and the development and severity of postoperative AKI. Their results suggested that increased baseline MPV values indicated an elevated risk for postoperative AKI, but this association did not remain statistically significant after adjusting for relevant clinical variables. To facilitate clinical application and avoid the effect of intraoperative platelet transfusion on postoperative MPV, we only considered and included baseline MPV as a potential risk factor without recording the perioperative change of MPV. Additionally, based on the results of our literature search, this is the first time MPV has been identified as a strong predictor of postoperative AKI in a broad range of cardiac surgery. Compared to the study by Abinaya et al. (28), where nearly one-third of patients had non-coronary cardiac procedures, our analysis expanded the cohort to include cardiac procedures such as heart transplantation, aortic surgery with CPB, and other complex combined procedures. MPV may serve as a significant biomarker with important clinical implications, yet its value is often overlooked and underestimated. However, the complex association between preoperative MPV, the role of platelets, and postoperative AKI needs to be further explored in future research. For instance, whether commonly used antiplatelet drugs in cardiac surgery patients affect MPV and the incidence of AKI could be a potential area for intervention.

Furthermore, our findings revealed a significant association between increased intraoperative cryoprecipitate transfusion volume and the risk of developing AKI (OR: 1.110, 95% CI: 1.050–1.173; *P* < 0.001). Transfusion is commonly administered during cardiac surgery and its detrimental effects are multifaceted, including a systemic inflammatory response that contributes to postoperative AKI development (29, 30). Cryoprecipitate, containing Factor VIII, Factor XIII, von Willebrand Factor, fibrinogen, and fibronectin, is primarily used to treat acquired hypofibrinogenemia in cardiac surgery. In Europe, there has been a gradual shift towards using fibrinogen concentrate due to its convenience in clinical application and concerns regarding viral transmission risks, although high-quality evidence in this area is still lacking (31). However, reports examining the association between intraoperative cryoprecipitate transfusion in adult cardiac surgery and postoperative AKI are scarce. Hinton et al. (32) analyzed data from the Medical Information Mart for Intensive Care (MIMIC) III and IV databases and found that cryoprecipitate administration after cardiac surgery was infrequent, and postoperative cryoprecipitate transfusion was not significantly associated with AKI (OR: 1.03, 99% CI 0.65–1.62, *P* = 0.876). Conversely, Jake et al. (33) conducted a study involving 119,132 eligible patients and concluded that postoperative cryoprecipitate transfusion was associated with a reduction in acute kidney injury (OR: 0.85, 99% CI, 0.73–0.98; *P* = 0.0037). A substantial amount of cryoprecipitate transfusion in cardiac surgery reflects excessive surgical bleeding, resulting in renal hypoperfusion and ischemia, which may be linked to AKI. With this consideration, INR, intraoperative bleeding volume, and the volume of transfused blood products were included in the multivariable logistic regression, and the results still showed a significant association between cryoprecipitate and AKI. To gain a deeper understanding of the underlying mechanism linking intraoperative cryoprecipitate transfusion and renal injury, further high-quality research is warranted. Optimizing perioperative transfusion management could have significant implications for postoperative renal outcomes.

Moreover, our study demonstrated a significant elevation in baseline BNP levels among patients with AKI compared to those without AKI. By examining the nonlinear relationship between baseline BNP levels and AKI occurrence following cardiac surgery, we discovered that the commonly used cutoff value for heart failure diagnosis (34) exhibited excellent predictive capabilities for AKI development. Heart failure often leads to venous congestion, which is associated with adverse renal events after surgery (35). In multivariable logistic regression, after adjusting for preoperative ejection fraction and cardiac chamber size, BNP remained significantly associated with AKI. Consistent with previous research, preoperative BNP levels emerged as a risk factor for AKI post-cardiac surgery (36). Our study encompassed a diverse population of patients undergoing cardiac surgery, including various surgical subtypes, thus validating the clinical applicability of BNP as a predictive tool. Optimizing perioperative organ function status and monitoring and adjusting BNP levels may provide crucial guidance in reducing the risk of AKI.

Furthermore, the findings of our study indicate that preoperative atrial fibrillation is a predictive factor for CS-AKI (OR: 2.183, 95% CI: 1.266–3.762; *P* = 0.005). Previous studies have also suggested a correlation between preoperative atrial fibrillation and adverse kidney events subsequent to cardiac surgery (35). AF represents the most prevalent heart rhythm disorder, and recent studies have consistently demonstrated a close association between AF and AKI (37, 38). Chan et al. (39) observed a significant five-fold increase in the incidence of AKI necessitating dialysis among 3,497,677 individuals hospitalized for AF between 2003 and 2012 in the United States. Li et al. (40) reported a significant association between preoperative atrial fibrillation and AKI diagnosed within 48 h to 7 days following on-pump cardiac surgery. The relationship between AF and AKI subsequent to cardiac surgery is complex, highlighting the need for improved clinical management of cardiac surgery patients with atrial fibrillation during the perioperative period.

## 5 Limitation

This study had several limitations that should be acknowledged. Firstly, it was a retrospective, single-center, observational case-control study, potentially limiting the generalizability of our findings to other settings. Future multi-center studies are necessary to validate our results across different healthcare environment, even in non-cardiac surgeries. Secondly, the retrospective nature of our study imposed potential bias. Reliance on medical records may introduce information bias. Unrecognized or undetermined confounders may also mediate the occurrence of kidney injury. Thirdly, in this study, the diagnosis and classification of AKI were solely based on creatinine levels and did not incorporate urine output criteria or the detection of early novel kidney injury biomarkers. Lastly, this study lacks the identification and research of long-term renal outcomes, such as CKD, as well as targeted prospective clinical intervention studies for the identified risk factors, necessitating further investigation in future research.

## 6 Conclusion

In summary, this study found that postoperative AKI was prevalent among patients undergoing cardiac surgery and was associated with higher in-hospital mortality, particularly in stages AKI-II and AKI-III. Several independent risk factors for postoperative AKI were identified in patients undergoing various types of cardiac surgeries. Notably, MPV and administration of cryoprecipitate may have new considerable predictive significance. The study also highlights the need for optimizing perioperative management to prevent or mitigate the impact of AKI, such as maintaining normal levels of lactate and other homeostatic factors, and optimizing the perioperative transfusion management. Meanwhile, our findings may guide the allocation of more healthcare resources before AKI patients develop more severe complications, leading to better clinical outcome.

## Data Availability

The original contributions presented in the study are included in the article/Supplementary Material, further inquiries can be directed to the corresponding author.

## Supplementary material

Supplementary Figure 1 Non-linear associations of BNP, INR, and Hb with AKI.

Supplementary Figure 2 Sensitivity analysis: Receiver-operating characteristic (ROC) curve for evaluating the discrimination performance of the logistic model in the study cohort.
