# Supplementary material for: The incidence, risk factors, and prognosis of acute kidney injury in patients after cardiac surgery
Source: Front Cardiovasc Med. 2024 Jul 16;11:1396889. doi: 10.3389/fcvm.2024.1396889 (PMC11286402; doi:10.3389/fcvm.2024.1396889)
Supplement: Supplementary file 1 [file Datasheet1.zip › Data Sheet 1_v1/Supplementary Table 1.pdf]

Supplementary Table 1. Demographic data of the total population, patients with or without AKI. AKI, acute kidney injury; ……

	Total (n = 868)	Non-AKI (n = 611)	AKI (n = 257)	*p value*
LA volume (mL), Median (IQR)	68.0 (46.0, 118.2)	64.0 (46.0, 107.0)	82.0 (50.0, 145.0)	< 0.001
RA volume (mL), Median (IQR)	35.0 (25.0, 53.0)	34.0 (25.0, 48.0)	37.0 (27.0, 63.0)	0.017
LV volume (mL), Median (IQR)	103.0 (76.0, 143.2)	102.0 (74.5, 139.0)	107.0 (79.0, 151.0)	0.096
RV volume (mL), Median (IQR)	23.0 (16.0, 33.0)	23.0 (16.0, 32.0)	25.0 (17.0, 35.0)	0.266
TB (μmol/L), Median (IQR)	13.6 (10.0, 18.1)	13.0 (9.9, 17.3)	14.8 (10.7, 20.9)	0.001
DB (μmol/L), Median (IQR)	4.7 (3.3, 6.9)	4.5 (3.2, 6.4)	5.3 (3.6, 8.1)	< 0.001
ALT u/L, Median (IQR)	21.0 (15.0, 32.0)	21.0 (15.0, 32.0)	21.0 (15.0, 32.0)	0.641
AST u/L, Median (IQR)	19.0 (16.0, 26.0)	19.0 (16.0, 24.0)	21.0 (16.0, 28.0)	0.002
BUN (mmol/L), Median (IQR)	6.1 (5.1, 7.5)	5.9 (5.0, 7.2)	6.6 (5.6, 8.4)	< 0.001
WBC (10^9/L), Median (IQR)	6.1 (5.0, 7.6)	6.1 (5.0, 7.4)	6.1 (5.0, 8.5)	0.111
Lym (10^9/L), Mean ± SD	1.7 ± 0.7	1.8 ± 0.7	1.5 ± 0.7	< 0.001
Neu (10^9/L), Median (IQR)	3.6 (2.7, 4.8)	3.6 (2.7, 4.6)	3.6 (2.8, 5.6)	0.009
NLR, Median (IQR)	2.1 (1.5, 3.0)	2.0 (1.5, 2.8)	2.4 (1.7, 3.9)	< 0.001
PLR, Median (IQR)	113.1 (86.6, 154.3)	111.7 (86.8, 145.5)	118.0 (84.6, 172.8)	0.123
SII, Median (IQR)	394.0 (263.0, 612.4)	386.8 (259.8, 576.3)	419.5 (273.6, 835.7)	0.016
RBC (10^9/L), Mean ± SD	4.4 ± 0.8	4.5 ± 0.7	4.3 ± 1.0	0.005
RDW (%), Median (IQR)	12.9 (12.4, 13.6)	12.8 (12.3, 13.4)	13.1 (12.5, 14.4)	< 0.001
D-dimer (mg/L), Median (IQR)	0.4 (0.3, 0.6)	0.3 (0.3, 0.5)	0.4 (0.3, 1.0)	< 0.001
**Intraoperative factors**				
Duration of anesthesia(min), Median (IQR)	280.0 (240.0, 325.0)	265.0 (230.0, 310.0)	305.0 (262.5, 377.5)	< 0.001
Nasopharyngeal temperature(°C), Median (IQR)	32.5 (31.9, 32.9)	32.5 (32.0, 32.9)	32.3 (31.7, 32.9)	< 0.001
Anal temperature(°C), Median (IQR)	33.2 (32.6, 33.8)	33.3 (32.8, 33.8)	33.0 (32.2, 33.5)	< 0.001

LA, left atrial; RA, right atrial; LV, left ventricular; RV, right ventricular; TB, total bilirubin; DB, direct bilirubin; ALT, alanine aminotransferase; AST, aspartate aminotransferase; BUN, blood urea nitrogen; WBC, white blood cell; Lym, lymphocyte; Neu, neutrophil; SII, systemic immune-inflammation index; NLR, neutrophil-to-lymphocyte ratio; PLR, platelet-to-lymphocyte ratio; RBC, red blood cell; RDW, red blood cell distribution width.
