# Supplementary material for: The incidence, risk factors, and prognosis of acute kidney injury in patients after cardiac surgery
Source: Front Cardiovasc Med. 2024 Jul 16;11:1396889. doi: 10.3389/fcvm.2024.1396889 (PMC11286402; doi:10.3389/fcvm.2024.1396889)
Supplement: Supplementary file 1 [file Datasheet1.zip › Data Sheet 1_v1/Supplementary Table 2 and 3.pdf]

Supplementary Table 2. Baseline characteristics of the patients with AKI of different stages. AKI, acute kidney injury……

	AKI Stage 1 (n = 179)	AKI Stage 2 (n = 39)	AKI Stage 3 (n = 39)	*p value*
Age (yr), Median (IQR)	62.0 (53.0, 70.0)	62.0 (51.0, 67.0)	63.0 (53.5, 69.5)	0.662
Gender, n (%)				0.863
Male	116 (64.8)	26 (66.7)	27 (69.2)	
Female	63 (35.2)	13 (33.3)	12 (30.8)	
BMI (kg/m^2), Median (IQR)	23.4 (21.6, 26.2)	23.2 (20.5, 27.2)	23.4 (21.3, 26.0)	0.993
ASA physical status, n (%)				0.755
ASA 1,2	21 (11.7)	3 (7.7)	5 (12.8)	
ASA ≥3	158 (88.3)	36 (92.3)	34 (87.2)	
Diabetes mellitus, n (%)	20 (11.2)	2 (5.1)	3 (7.7)	0.568
Hypertension n (%)	32 (17.9)	12 (30.8)	8 (20.5)	0.192
Chronic liver disease, n (%)	0 (0)	1 (2.6)	1 (2.6)	0.096
Atrial fibrillation, n (%)	40 (22.3)	12 (30.8)	5 (12.8)	0.161
Preoperative ECMO/IABP/or both support, n (%)	1 (0.6)	3 (7.7)	2 (5.1)	0.01
EF (%), Median (IQR)	58.0 (50.0, 65.0)	56.0 (45.5, 66.0)	53.0 (44.0, 63.0)	0.504
LA volume (mL), Median (IQR)	94.0 (60.0, 147.5)	101.5 (65.5, 154.0)	68.0 (53.0, 164.0)	0.499
RA volume (mL), Median (IQR)	39.0 (29.0, 65.0)	51.0 (32.0, 70.0)	41.0 (31.0, 70.0)	0.504
LV volume (mL), Median (IQR)	110.0 (87.0, 159.0)	142.0 (96.2, 181.5)	114.0 (78.0, 148.0)	0.382
RV volume (mL), Median (IQR)	26.0 (18.0, 38.0)	28.0 (22.2, 38.5)	25.0 (20.0, 34.0)	0.443
TB (μmol/L), Median (IQR)	14.2 (10.0, 20.0)	17.4 (11.7, 22.5)	17.0 (11.4, 24.8)	0.069
DB (μmol/L), Median (IQR)	5.1 (3.5, 7.4)	6.8 (4.1, 11.0)	5.7 (3.8, 9.9)	0.246
Albumin (g/L), Median (IQR)	40.0 (38.0, 43.0)	41.0 (38.5, 43.0)	39.0 (37.0, 41.0)	0.081
ALT u/L, Median (IQR)	20.5 (15.0, 32.0)	25.0 (18.5, 32.5)	20.0 (15.0, 33.0)	0.323
AST u/L, Median (IQR)	21.0 (16.0, 27.0)	23.5 (18.0, 28.0)	20.0 (17.2, 33.2)	0.409
BUN (mmol/L), Median (IQR)	6.6 (5.5, 8.3)	6.7 (6.2, 9.6)	6.5 (5.6, 8.7)	0.323
Cr (μmol/L), Median (IQR)	78.0 (64.0, 96.5)	81.0 (66.5, 87.0)	75.0 (65.5, 99.5)	0.965
Estimated glomerular filtration rate (ml/(min*1.73 m^2)), Median (IQR)	86.3 (71.3, 98.6)	88.3 (69.7, 99.9)	93.3 (65.9, 109.1)	0.738
WBC (10^9/L), Median (IQR)	6.0 (5.1, 8.2)	6.4 (5.1, 10.2)	6.0 (4.7, 8.7)	0.627
Lym (10^9/L), Mean ± SD	1.5 (1.1, 1.9)	1.4 (0.9, 1.8)	1.6 (0.8, 2.0)	0.446
Neu (10^9/L), Median (IQR)	3.6 (2.9, 5.2)	3.9 (3.0, 7.9)	3.7 (2.7, 7.1)	0.609
NLR, Median (IQR)	2.3 (1.7, 3.5)	2.7 (1.7, 7.1)	2.3 (1.5, 11.2)	0.559
PLR, Median (IQR)	116.9 (86.9, 160.8)	144.7 (79.8, 195.7)	122.8 (78.6, 190.7)	0.436
SII, Median (IQR)	415.7 (286.7, 688.6)	565.8 (242.3, 1507.1)	398.9 (213.6, 1677.9)	0.627
RBC (10^9/L), Mean ± SD	4.3 (3.9, 4.8)	4.3 (3.9, 4.8)	4.2 (3.7, 4.5)	0.627
MPV (fL), Mean ± SD	11.2 (10.5, 12.2)	11.5 (10.5, 12.5)	11.2 (10.3, 12.8)	0.978
Platelet count (10^9/L), Median (IQR)	174.0 (137.5, 226.0)	160.0 (120.2, 215.2)	158.5 (122.2, 216.5)	0.412
Hemoglobin (g/L), Mean ± SD	130.0 (117.5, 145.0)	129.5 (111.5, 144.8)	127.0 (111.2, 140.0)	0.825
RDW (%), Median (IQR)	13.1 (12.5, 14.2)	13.7 (12.5, 15.0)	12.9 (12.5, 14.0)	0.275
BNP (pg/ml), Median (IQR)	180.4 (87.8, 454.3)	208.9 (113.9, 539.6)	123.8 (86.7, 281.7)	0.215
INR, Median (IQR)	1.1 (1.0, 1.2)	1.1 (1.0, 1.2)	1.1 (1.0, 1.2)	0.479
D-dimer (mg/L), Median (IQR)	0.4 (0.3, 0.8)	0.5 (0.3, 1.3)	0.5 (0.3, 1.7)	0.312
**Surgery-related characteristics**				
Emergency operation, n (%)	25 (14)	12 (30.8)	12 (30.8)	0.007
Off pump, n (%)	4 (2.2)	1 (2.6)	2 (5.1)	0.591
Surgical types, n (%)				0.15
CABG only	28 (15.6)	2 (5.1)	3 (7.7)	
Single-Valve replacement only	17 (9.5)	4 (10.3)	2 (5.1)	

Multiple-Valve replacement surgery only	30 (16.8)	7 (17.9)	5 (12.8)	
Combined CABG-valve procedure	11 (6.1)	2 (5.1)	0 (0)	
Aortic procedure	33 (18.4)	11 (28.2)	13 (33.3)	
Heart transplantation	6 (3.4)	4 (10.3)	4 (10.3)	
Others	54 (30.2)	9 (23.1)	12 (30.8)	
Aortic dissection surgery, n (%)	21 (11.7)	6 (15.4)	8 (20.5)	0.329
**Intraoperative factors**				
Intraoperative crystalloid infusion(ml), Median (IQR)	1200.0 (1100.0, 1600.0)	1200.0 (1100.0, 1710.0)	1300.0 (1100.0, 1750.0)	0.212
Intraoperative transfusion volume(ml), Median (IQR)				
Total	0.0 (0.0, 14.0)	10.0 (0.0, 21.0)	15.0 (0.0, 25.0)	0.005
Erythrocytes	0.0 (0.0, 400.0)	0.0 (0.0, 400.0)	400.0 (0.0, 800.0)	0.051
Plasma	0.0 (0.0, 400.0)	0.0 (0.0, 400.0)	0.0 (0.0, 400.0)	0.192
Platelet	0.0 (0.0, 0.0)	0.0 (0.0, 10.0)	0.0 (0.0, 10.0)	0.031
Cryoprecipitate	0.0 (0.0, 10.0)	10.0 (0.0, 10.0)	10.0 (0.0, 10.0)	0.002
Intraoperative blood loss(ml), Median (IQR)	300.0 (200.0, 500.0)	300.0 (200.0, 500.0)	300.0 (200.0, 500.0)	0.528
Intraoperative urine output(ml), Median (IQR)	1000.0 (725.0, 1500.0)	1050.0 (800.0, 1675.0)	1100.0 (700.0, 2000.0)	0.588
Duration of surgery(min), Median (IQR)	260.0 (220.0, 312.5)	300.0 (227.5, 377.5)	300.0 (237.5, 372.5)	0.023
Duration of anesthesia(min), Median (IQR)	300.0 (260.0, 355.0)	335.0 (273.0, 430.0)	340.0 (265.0, 415.0)	0.055
DHCA, n (%)	2 (1.1)	0 (0)	3 (8.1)	0.047
Nasopharyngeal temperature(℃), Median (IQR)	32.4 (31.9, 32.9)	32.0 (31.5, 32.6)	32.1 (27.7, 32.6)	0.042
Anal temperature(℃), Median (IQR)	33.1 (32.4, 33.5)	32.8 (32.1, 33.5)	32.9 (28.6, 33.2)	0.066
Minimum intraoperative Hb level(g/L), Median (IQR)	6.8 (5.9, 8.2)	7.3 (6.1, 8.6)	6.8 (6.0, 7.7)	0.378
Minimum intraoperative Hct level(%), Median (IQR)	21.5 (19.0, 25.0)	23.0 (18.6, 27.5)	21.0 (18.0, 24.0)	0.339
Minimum intraoperative PaO_2(mmHg), Median (IQR)	260.0 (146.8, 309.0)	234.0 (139.2, 268.0)	228.0 (151.2, 299.8)	0.401
Maximum intraoperative lactate level(mmol/L), Median (IQR)	3.5 (2.5, 5.1)	4.6 (2.2, 6.6)	4.6 (2.6, 6.6)	0.188

BMI, body mass index; ASA, American Society of Anesthesiologists; ECMO, extra-corporeal membrane oxygenation; IABP, intra-aortic balloon pump; EF, ejection fraction; LA, left atrial; RA, right atrial; LV, left ventricular; RV, right ventricular; TB, total bilirubin; DB, direct bilirubin; ALT, alanine aminotransferase; AST, aspartate aminotransferase; BUN, blood urea nitrogen; Cr, creatinine; WBC, white blood cell; Lym, lymphocyte; Neu, neutrophil; SII, systemic immune-inflammation index; NLR, neutrophil-to-lymphocyte ratio; PLR, platelet-to-lymphocyte ratio; MPV, mean platelet volume; RBC, red blood cell; RDW, red blood cell distribution width; INR, international normalized ratio; BNP, brain natriuretic peptide; CABG, coronary artery bypass graft surgery; DHCA, deep hypothermic circulatory arrest; Hb, hemoglobin; Hct, hematocrit; PaO2, partial pressure of oxygen in arterial blood.

Supplementary Table 3. Postoperative outcomes of the patients with AKI of different stages. AKI, acute kidney injury……

	AKI Stage 1 (n = 179)	AKI Stage 2 (n = 39)	AKI Stage 3 (n = 39)	*p value*
**Postoperative outcomes**				

Duration of mechanical ventilation in ICU(h), Median (IQR)	17.5 (8.0, 22.3)	21.9 (18.0, 59.8)	22.8 (15.5, 62.5)	< 0.001
>24h	35 (19.6)	19 (48.7)	19 (48.7)	< 0.001
>48h	12 (6.7)	12 (30.8)	15 (38.5)	< 0.001
Reintubation, n (%)	7 (3.9)	4 (10.3)	6 (15.4)	0.025
Tracheostomy, n (%)	4 (2.2)	3 (7.7)	7 (17.9)	< 0.001
Maximum postoperative PCT level(ng/ml), Median (IQR)	2.4 (0.7, 6.1)	7.4 (3.4, 12.0)	17.8 (5.6, 38.8)	< 0.001
Initiation of CRRT, n (%)	3 (1.7)	2 (5.1)	20 (51.3)	< 0.001
Cardiac arrest, n (%)	4 (2.2)	0 (0)	5 (12.8)	0.011
Redo surgery, n (%)	8 (4.5)	6 (15.8)	10 (25.6)	< 0.001
Postoperative ECMO/IABP/or both support, n (%)	10 (5.6)	13 (33.3)	18 (46.2)	< 0.001
LOS-ICU(d), Median (IQR)	4.0 (3.0, 6.0)	8.0 (4.0, 14.0)	10.0 (6.0, 22.5)	< 0.001
LOS(d), Median (IQR)	23.0 (18.0, 29.0)	26.0 (21.0, 34.0)	24.0 (18.5, 39.0)	0.109
Postoperative LOS(d), Median (IQR)	15.0 (12.0, 21.0)	19.0 (15.5, 26.5)	18.0 (13.0, 29.0)	0.024
In-hospital mortality, n (%)	9 (5)	7 (17.9)	17 (43.6)	< 0.001
30-day mortality, n (%)	7 (3.9)	7 (17.9)	14 (35.9)	< 0.001

ICU, intensive care unit; PCT = procalcitonin; CRRT, continuous renal replacement therapy; LOS = length of stay; LOS-ICU, length of stay in ICU.
