# Supplementary material for: The incidence, risk factors, and prognosis of acute kidney injury in patients after cardiac surgery
Source: Front Cardiovasc Med. 2024 Jul 16;11:1396889. doi: 10.3389/fcvm.2024.1396889 (PMC11286402; doi:10.3389/fcvm.2024.1396889)
Supplement: Supplementary file 1 [file Datasheet1.zip › Data Sheet 1_v1/Supplementary Table 4.pdf]

Supplementary Table 4. Univariate analysis of risk factors for AKI after cardiac surgery. AKI, acute kidney injury……

Predictors	Odds Ratio	95% confidence interval		*P* value
		Lower	Upper	
Age, per 5-yr increase	1.16	1.09	1.23	<0.001
ASA≥3	4.06	2.636	6.18	<0.001
Atrial fibrillation	2.52	1.7	3.74	<0.001
Cr	1.02	1.01	1.03	<0.001
eGFR	0.98	0.98	0.99	<0.001
Lym	0.57	0.45	0.71	<0.001
Neu	1.13	1.07	1.2	<0.001
RBC	0.73	0.58	0.9	0.004
Hb categories	0.69	0.56	0.85	<0.001
MPV	1.2	1.07	1.35	0.002
RDW	1.26	1.15	1.39	<0.001
TB	1.03	1.01	1.05	0.001
DB	1.07	1.04	1.1	<0.001
Albumin	0.92	0.88	0.95	<0.001
WBC	1.09	1.04	1.15	0.001
NLR	1.11	1.07	1.15	<0.001
PLR	1.00	1.00	1.00	0.017
SII	1.00	1.00	1.00	<0.001
Platelet	1	0.99	1	0.001
D-dimer	1.21	1.11	1.31	<0.001
LA volume	1.01	1.00	1.01	<0.001
RA volume	1.01	1.00	1.01	0.001
LV volume	1.00	1.00	1.01	<0.001
INR categories	2.94	2.17	3.99	<0.001
BNP categories	1.95	1.6	2.38	<0.001
EF	0.98	0.97	0.99	0.002
On pump surgery	2.64	1.17	5.95	0.02
Surgical types	1.14	1.07	1.213	<0.001
Aortic dissection surgery	6.72	3.55	12.73	<0.001
Intraoperative platelet transfusion volume	1.16	1.11	1.20	<0.001
Intraoperative cryoprecipitate transfusion volume	1.16	1.12	1.2	<0.001
Nasopharyngeal temperature	0.8	0.74	0.87	<0.001
Anal temperature	0.72	0.66	0.79	<0.001
Minimum intraoperative Hb level	0.89	0.82	0.96	0.004
Minimum intraoperative Hct level	0.95	0.92	0.97	<0.001
Maximum intraoperative lactate level	1.48	1.35	1.61	<0.001
Intraoperative crystalloid infusion	1.00	1.00	1.00	<0.001
Intraoperative erythrocyte transfusion volume	1.00	1.00	1.00	<0.001
Intraoperative plasma transfusion volume	1.00	1.00	1.00	<0.001
Intraoperative total transfusion volume	1.08	1.06	1.09	<0.001
Intraoperative blood loss	1.00	1.00	1.00	0.002
Intraoperative urine output	1.00	1.00	1.00	0.004
Duration of surgery	1.01	1.01	1.01	<0.001
Duration of anesthesia	1.01	1.01	1.01	<0.001

ASA, American Society of Anesthesiologists; Cr, creatinine; eGFR=estimated glomerular filtration rate; Lym, lymphocyte; Neu, neutrophil; RBC, red blood cell; Hb, hemoglobin; MPV, mean platelet volume; RDW, red blood cell distribution width; TB, total bilirubin; DB, direct bilirubin; WBC, white blood cell; NLR, neutrophil-to-lymphocyte ratio; PLR, platelet-to-lymphocyte ratio; SII, systemic immune-inflammation index; LA, left atrial; RA, right atrial; LV, left ventricular; RV, right ventricular; Hb, hemoglobin; INR, international normalized ratio; BNP, brain natriuretic peptide; EF, ejection fraction; Hct, hematocrit.
