# Supplementary material for: The incidence, risk factors, and prognosis of acute kidney injury in patients after cardiac surgery
Source: Front Cardiovasc Med. 2024 Jul 16;11:1396889. doi: 10.3389/fcvm.2024.1396889 (PMC11286402; doi:10.3389/fcvm.2024.1396889)
Supplement: Supplementary file 1 [file Datasheet1.zip › Data Sheet 1_v1/Supplementary Table 5 and 6.pdf]

Supplementary Table 5. Baseline characteristics of total population, patients with or without AKI undergoing the on-pump surgery, excluding heart transplantation. AKI, acute kidney injury; ……

	Total (n = 795)	Non-AKI (n = 559	AKI (n = 236)	*p value*
Age (yr), Median (IQR)	58.0 (50.0, 67.0)	57.0 (48.0, 65.5)	63.0 (53.0, 69.2)	< 0.001
Gender, n (%)				0.396
Male	501 (63.0)	347 (62.1)	154 (65.3)	
Female	294 (37.0)	212 (37.9)	82 (34.7)	
BMI (kg/m^2), Median (IQR)	23.5 (21.6, 26.0)	23.6 (21.6, 25.9)	23.4 (21.4, 26.4)	0.891
ASA physical status, n (%)				< 0.001
ASA 1,2	221 (27.8)	195 (34.9)	26 (11)	
ASA ≥3	574 (72.2)	364 (65.1)	210 (89)	
Diabetes mellitus, n (%)	67 (8.4)	44 (7.9)	23 (9.7)	0.385
Hypertension n (%)	151 (19.0)	100 (17.9)	51 (21.6)	0.222
Chronic liver disease, n (%)	12 (1.5)	10 (1.8)	2 (0.8)	0.525
Atrial fibrillation, n (%)	119 (15.0)	62 (11.1)	57 (24.2)	< 0.001
Preoperative ECMO/IABP/or both support, n (%)	1 (0.1)	1 (0.2)	0 (0)	1
EF (%), Median (IQR)	60.0 (52.0, 66.0)	60.0 (53.0, 67.0)	58.0 (50.0, 65.0)	0.008
LA volume (mL), Median (IQR)	75.0 (53.0, 123.2)	69.0 (51.0, 112.5)	92.0 (60.0, 148.0)	< 0.001
RA volume (mL), Median (IQR)	37.0 (27.0, 55.0)	36.0 (27.0, 51.0)	42.0 (29.0, 65.0)	0.001
LV volume (mL), Median (IQR)	107.0 (81.0, 147.0)	106.0 (79.0, 145.0)	112.0 (87.0, 155.0)	0.012
RV volume (mL), Median (IQR)	25.0 (18.0, 34.0)	23.0 (17.0, 33.0)	27.0 (19.0, 36.0)	0.054
TB (µmol/L), Median (IQR)	13.5 (10.0, 18.1)	13.1 (9.9, 17.6)	14.6 (10.6, 20.5)	0.009
DB (µmol/L), Median (IQR)	4.7 (3.3, 6.8)	4.4 (3.2, 6.4)	5.2 (3.5, 7.7)	< 0.001
Albumin (g/L), Median (IQR)	41.0 (39.0, 44.0)	41.0 (39.0, 44.0)	40.0 (38.0, 43.0)	< 0.001
ALT u/L, Median (IQR)	21.0 (14.2, 31.8)	21.0 (14.0, 31.0)	21.0 (15.0, 32.0)	0.581
AST u/L, Median (IQR)	19.0 (16.0, 26.0)	19.0 (16.0, 24.0)	21.0 (16.0, 28.0)	0.002
BUN (mmol/L), Median (IQR)	6.1 (5.0, 7.4)	5.9 (5.0, 7.1)	6.6 (5.5, 8.1)	< 0.001
Cr (µmol/L), Median (IQR)	73.0 (60.0, 84.0)	71.0 (60.0, 81.0)	76.5 (64.0, 93.2)	< 0.001
Estimated glomerular filtration rate (ml/(min*1.73 m^2)), Median (IQR)	95.6 (79.3, 110.2)	98.8 (81.8, 112.4)	88.8 (71.6, 100.5)	< 0.001
WBC (10^9/L), Median (IQR)	6.0 (4.9, 7.6)	6.0 (4.9, 7.4)	6.0 (5.0, 8.3)	0.169
Lym (10^9/L), Mean ± SD	1.6 (1.3, 2.1)	1.8 (1.3, 2.2)	1.5 (1.1, 1.9)	< 0.001
Neu (10^9/L), Median (IQR)	3.5 (2.7, 4.8)	3.5 (2.6, 4.5)	3.6 (2.8, 5.4)	0.007
NLR, Median (IQR)	2.1 (1.5, 2.9)	2.0 (1.5, 2.7)	2.3 (1.7, 3.7)	< 0.001
PLR, Median (IQR)	112.5 (86.6, 151.2)	109.9 (86.5, 144.3)	118.0 (87.2, 171.6)	0.072
SII, Median (IQR)	384.5 (256.4, 602.7)	372.6 (255.4, 554.6)	417.6 (272.0, 832.5)	0.009
RBC (10^9/L), Mean ± SD	4.4 (4.1, 4.8)	4.4 (4.1, 4.8)	4.3 (3.9, 4.8)	< 0.001
MPV (fL), Mean ± SD	11.1 (10.4, 12.0)	11.0 (10.3, 12.0)	11.2 (10.4, 12.3)	0.043
Platelet (10^9/L), Median (IQR)	188.0 (147.0, 228.0)	193.5 (155.2, 230.0)	169.0 (131.2, 223.5)	< 0.001
Hemoglobin (g/L), Mean ± SD	135.0 (124.0, 147.0)	136.0 (126.0, 147.0)	129.5 (117.0, 145.0)	< 0.001
RDW (%), Median (IQR)	12.9 (12.4, 13.6)	12.8 (12.3, 13.4)	13.0 (12.5, 14.1)	< 0.001

BNP (pg/ml), Median (IQR)	115.3 (49.0, 279.4)	88.1 (39.5, 223.8)	172.0 (89.0, 403.5)	< 0.001
INR, Median (IQR)	1.0 (1.0, 1.1)	1.0 (1.0, 1.1)	1.1 (1.0, 1.2)	< 0.001
D-dimer (mg/L), Median (IQR)	0.4 (0.3, 0.6)	0.3 (0.3, 0.5)	0.4 (0.3, 0.9)	< 0.001
**Surgery-related characteristics**				
Emergency operation, n (%)	60 (7.6)	23 (4.1)	37 (15.7)	< 0.001
Surgical types, n (%)				< 0.001
CABG only	136 (17.1)	109 (19.5)	27 (11.4)	
Single-Valve replacement only	137 (17.2)	114 (20.4)	23 (9.7)	
Multiple-Valve replacement surgery only	117 (14.7)	75 (13.4)	42 (17.8)	
Combined CABG-valve procedure	31 (3.9)	18 (3.2)	13 (5.5)	
Aortic procedure	122 (15.3)	65 (11.6)	57 (24.2)	
Others	252 (31.7)	178 (31.8)	74 (31.4)	
Aortic dissection surgery, n (%)	49 (6.2)	14 (2.5)	35 (14.8)	< 0.001
**Intraoperative factors**				
Intraoperative crystalloid infusion(ml), Median (IQR)	1100.0 (1100.0, 1310.0)	1100.0 (1100.0, 1220.0)	1200.0 (1100.0, 1600.0)	< 0.001
Total intraoperative transfusion volume(u), Median (IQR)	0.0 (0.0, 7.0)	0.0 (0.0, 0.0)	4.0 (0.0, 18.2)	< 0.001
Intraoperative transfusion volume(ml), Median (IQR)				
Erythrocytes	0.0 (0.0, 0.0)	0.0 (0.0, 0.0)	0.0 (0.0, 400.0)	< 0.001
Plasma	0.0 (0.0, 200.0)	0.0 (0.0, 0.0)	0.0 (0.0, 400.0)	< 0.001
Platelet	0.0 (0.0, 0.0)	0.0 (0.0, 0.0)	0.0 (0.0, 0.0)	< 0.001
Cryoprecipitate	0.0 (0.0, 0.0)	0.0 (0.0, 0.0)	0.0 (0.0, 10.0)	< 0.001
Intraoperative blood loss(ml), Median (IQR)	300.0 (200.0, 500.0)	300.0 (200.0, 400.0)	300.0 (200.0, 500.0)	0.001
Intraoperative urine output(ml), Median (IQR)	1000.0 (800.0, 1500.0)	1000.0 (800.0, 1400.0)	1000.0 (800.0, 1600.0)	0.078
Duration of surgery(min), Median (IQR)	235.0 (200.0, 285.0)	225.0 (190.0, 265.0)	265.0 (220.0, 330.0)	< 0.001
Duration of anesthesia(min), Median (IQR)	280.0 (240.0, 325.0)	265.0 (230.0, 310.0)	305.0 (260.0, 375.0)	< 0.001
DHCA, n (%)	9 (1.1)	5 (0.9)	4 (1.7)	0.462
Nasopharyngeal temperature(℃), Median (IQR)	32.4 (31.9, 32.9)	32.5 (32.0, 32.9)	32.3 (31.7, 32.9)	0.002
Anal temperature(℃), Median (IQR)	33.2 (32.6, 33.7)	33.3 (32.7, 33.8)	33.0 (32.2, 33.5)	< 0.001
Minimum intraoperative Hb level(g/L), Median (IQR)	7.1 (6.1, 8.4)	7.2 (6.1, 8.5)	6.8 (5.9, 8.2)	0.039
Minimum intraoperative Hct level (%), Median (IQR)	22.9 (19.0, 26.0)	23.0 (20.0, 26.0)	21.9 (19.0, 25.0)	0.002
Minimum intraoperative PaO_2(mmHg), Median (IQR)	258.0 (164.5, 307.0)	259.5 (170.0, 308.5)	252.5 (146.2, 307.0)	0.261
Maximum intraoperative lactate level(mmol/L), Median (IQR)	2.8 (2.0, 3.9)	2.5 (1.9, 3.4)	3.5 (2.5, 5.4)	< 0.001

BMI, body mass index; ASA, American Society of Anesthesiologists; ECMO, extra-corporeal membrane oxygenation; IABP, intra-aortic balloon pump; EF, ejection fraction; LA, left atrial; RA, right atrial; LV, left ventricular; RV, right ventricular; TB,

total bilirubin; DB, direct bilirubin; ALT, alanine aminotransferase; AST, aspartate aminotransferase; BUN, blood urea nitrogen; Cr, creatinine; WBC, white blood cell; Lym, lymphocyte; Neu, neutrophil; SII, systemic immune-inflammation index; NLR, neutrophil-to-lymphocyte ratio; PLR, platelet-to-lymphocyte ratio; MPV, mean platelet volume; RBC, red blood cell; RDW, red blood cell distribution width; INR, international normalized ratio; BNP, brain natriuretic peptide; CABG, coronary artery bypass graft surgery; DHCA, deep hypothermic circulatory arrest; Hb, hemoglobin; Hct, hematocrit; PaO2, partial pressure of oxygen in arterial blood.

Supplementary Table 6. Postoperative outcomes of the total population, patients with or without AKI undergoing the on-pump surgery, excluding heart transplantation. AKI, acute kidney injury……

	Total (n = 795)	Non-AKI (n = 559)	AKI (n = 236)	*p value*
**Postoperative outcomes**				
Duration of mechanical ventilation in ICU(h), Median (IQR)	9.5 (4.5, 20.0)	6.5 (4.2, 17.5)	19.0 (9.0, 24.0)	< 0.001
>24h	105 (13.2)	39 (7)	66 (28)	< 0.001
>48h	41 (5.2)	9 (1.6)	32 (13.6)	< 0.001
Reintubation, n (%)	23 (2.9)	7 (1.3)	16 (6.8)	< 0.001
Tracheostomy, n (%)	13 (1.6)	0 (0)	13 (5.5)	< 0.001
Maximum postoperative PCT level(ng/ml), Median (IQR)	1.2 (0.5, 4.1)	0.9 (0.4, 1.9)	3.7 (1.1, 9.4)	< 0.001
Initiation of CRRT, n (%)	21 (2.6)	0 (0)	21 (8.9)	< 0.001
Cardiac arrest, n (%)	10 (1.3)	2 (0.4)	8 (3.4)	0.001
Redo surgery, n (%)	44 (5.5)	23 (4.1)	21 (9)	0.006
Postoperative ECMO/IABP/or both support, n (%)	39 (4.9)	8 (1.4)	31 (13.1)	< 0.001
LOS-ICU(d), Median (IQR)	3.0 (2.0, 4.0)	2.0 (1.0, 3.0)	4.0 (3.0, 8.0)	< 0.001
LOS(d), Median (IQR)	19.0 (15.0, 25.0)	17.0 (14.0, 23.0)	23.0 (18.0, 30.0)	< 0.001
Postoperative LOS(d), Median (IQR)	13.0 (10.0, 18.0)	12.0 (9.0, 16.0)	16.0 (12.0, 22.0)	< 0.001
In-hospital mortality, n (%)	29 (3.6)	1 (0.2)	28 (11.9)	< 0.001
30-day mortality, n (%)	23 (2.9)	0 (0)	23 (9.7)	< 0.001

ICU, intensive care unit; PCT = procalcitonin; CRRT, continuous renal replacement therapy; LOS = length of stay; LOS-ICU, length of stay in ICU.
