# Supplementary material for: The incidence, risk factors, and prognosis of acute kidney injury in patients after cardiac surgery
Source: Front Cardiovasc Med. 2024 Jul 16;11:1396889. doi: 10.3389/fcvm.2024.1396889 (PMC11286402; doi:10.3389/fcvm.2024.1396889)
Supplement: Supplementary file 1 [file Datasheet1.zip › Data Sheet 1_v1/Supplementary Table 7 and 8.pdf]

Supplementary Table 7. Baseline characteristics of the patients undergoing on-pump surgery excluding heart transplantation with AKI of different stages. AKI, acute kidney injury……

	AKI Stage 1 (n = 169)	AKI Stage 2 (n = 34)	AKI Stage 3 (n = 33)	*p value*
Age (yr), Median (IQR)	63.0 (53.0, 70.0)	62.0 (52.2, 67.0)	63.0 (54.0, 70.0)	0.716
Gender, n (%)				0.621
Male	108 (63.9)	22 (64.7)	24 (72.7)	
Female	61 (36.1)	12 (35.3)	9 (27.3)	
BMI (kg/m^2), Median (IQR)	23.4 (21.5, 26.4)	23.2 (20.8, 27.6)	23.5 (21.0, 25.5)	0.951
ASA physical status, n (%)				0.95
ASA 1,2	19 (11.2)	3 (8.8)	4 (12.1)	
ASA ≥3	150 (88.8)	31 (91.2)	29 (87.9)	
Diabetes mellitus, n (%)	18 (10.7)	2 (5.9)	3 (9.1)	0.798
Hypertension n (%)	31 (18.3)	12 (35.3)	8 (24.2)	0.084
Chronic liver disease, n (%)	0 (0)	1 (2.9)	1 (3)	0.086
Atrial fibrillation, n (%)	40 (23.7)	12 (35.3)	5 (15.2)	0.151
Preoperative ECMO/IABP/or both support, n (%)	0 (0)	0 (0)	0 (0)	1
EF (%), Median (IQR)	58.0 (51.0, 65.0)	58.0 (50.0, 67.0)	55.5 (47.8, 63.2)	0.878
LA volume (mL), Median (IQR)	94.5 (60.0, 147.8)	99.0 (68.0, 158.5)	70.5 (52.8, 152.0)	0.452
RA volume (mL), Median (IQR)	38.0 (29.0, 64.5)	51.0 (33.0, 70.0)	39.5 (28.0, 55.8)	0.451
LV volume (mL), Median (IQR)	110.0 (87.0, 155.5)	141.0 (96.5, 176.5)	110.0 (82.0, 147.2)	0.48
RV volume (mL), Median (IQR)	26.0 (18.0, 37.8)	28.0 (22.5, 38.0)	24.5 (20.0, 32.2)	0.397
TB (μmol/L), Median (IQR)	14.1 (9.9, 19.4)	16.9 (11.9, 22.2)	16.8 (11.2, 23.8)	0.114
DB (μmol/L), Median (IQR)	5.0 (3.5, 7.3)	6.7 (4.2, 10.4)	5.2 (3.6, 9.3)	0.355
Albumin (g/L), Median (IQR)	40.0 (38.0, 43.0)	41.0 (39.0, 42.8)	39.0 (37.0, 41.0)	0.209
ALT u/L, Median (IQR)	20.0 (15.0, 32.0)	25.5 (19.0, 32.8)	20.0 (14.0, 32.0)	0.239
AST u/L, Median (IQR)	20.5 (16.0, 27.0)	24.0 (18.0, 28.0)	20.0 (15.0, 28.2)	0.371
BUN (mmol/L), Median (IQR)	6.5 (5.5, 8.1)	6.8 (6.4, 9.6)	6.4 (5.5, 7.8)	0.25
Cr (μmol/L), Median (IQR)	77.0 (64.0, 95.0)	78.5 (65.0, 85.8)	75.0 (66.0, 99.0)	0.975
Estimated glomerular filtration rate (ml/(min*1.73 m^2)), Median (IQR)	87.8 (71.6, 98.7)	89.7 (79.3, 100.2)	93.3 (69.5, 108.8)	0.721
WBC (10^9/L), Median (IQR)	6.0 (5.0, 8.0)	6.3 (5.0, 10.3)	6.0 (4.7, 8.0)	0.686
Lym (10^9/L), Mean ± SD	1.5 (1.1, 1.9)	1.4 (1.0, 1.7)	1.6 (0.9, 2.0)	0.43
Neu (10^9/L), Median (IQR)	3.5 (2.8, 5.1)	3.9 (2.8, 8.2)	3.7 (2.8, 5.6)	0.649
NLR, Median (IQR)	2.3 (1.7, 3.5)	2.8 (1.7, 7.2)	2.3 (1.5, 9.5)	0.513
PLR, Median (IQR)	116.9 (87.4, 158.1)	145.7 (96.4, 197.7)	118.4 (79.0, 187.2)	0.375
SII, Median (IQR)	414.5 (285.9, 690.8)	566.8 (239.5, 1629.9)	398.9 (198.5, 1081.0)	0.639
RBC (10^9/L), Mean ± SD	4.3 (3.9, 4.8)	4.4 (3.9, 4.8)	4.3 (3.8, 4.5)	0.897
MPV (fL), Mean ± SD	11.2 (10.5, 12.2)	11.2 (10.4, 12.6)	11.2 (10.3, 12.4)	0.988
Platelet count (10^9/L), Median (IQR)	174.0 (137.0, 226.0)	159.0 (117.0, 219.0)	158.0 (124.0, 219.0)	0.518
Hemoglobin (g/L), Mean ± SD	130.0 (117.0, 145.0)	130.0 (117.0, 146.0)	128.0 (117.2, 145.0)	0.966
RDW (%), Median (IQR)	13.0 (12.5, 14.1)	13.5 (12.5, 14.7)	12.8 (12.5, 13.6)	0.265
BNP (pg/ml), Median (IQR)	180.6 (89.1, 446.7)	186.2 (112.7, 362.9)	123.8 (86.7, 266.2)	0.414
INR, Median (IQR)	1.1 (1.0, 1.2)	1.1 (1.0, 1.2)	1.1 (1.0, 1.2)	0.494
D-dimer (mg/L), Median (IQR)	0.4 (0.3, 0.8)	0.5 (0.3, 1.0)	0.4 (0.3, 1.2)	0.684
**Surgery-related characteristics**				
Emergency operation, n (%)	147 (87.5)	26 (76.5)	25 (75.8)	0.096
Surgical types, n (%)				0.122
CABG only	25 (14.8)	1 (2.9)	1 (3)	
Single-Valve replacement only	17 (10.1)	4 (11.8)	2 (6.1)	

Multiple-Valve replacement surgery only	30 (17.8)	7 (20.6)	5 (15.2)	
Combined CABG-valve procedure	11 (6.5)	2 (5.9)	0 (0)	
Aortic procedure	33 (19.5)	11 (32.4)	13 (39.4)	
Others	53 (31.4)	9 (26.5)	12 (36.4)	
Aortic dissection surgery, n (%)	21 (12.4)	6 (17.6)	8 (24.2)	0.158
**Intraoperative factors**				
Intraoperative crystalloid infusion(ml), Median (IQR)	1120.0 (1100.0, 1600.0)	1200.0 (1100.0, 1675.0)	1200.0 (1100.0, 1700.0)	0.139
Total intraoperative transfusion volume(u), Median (IQR)	0.0 (0.0, 15.0)	5.0 (0.0, 20.0)	16.0 (0.0, 27.0)	0.019
Intraoperative transfusion volume(ml), Median (IQR)				
Erythrocytes	0.0 (0.0, 400.0)	0.0 (0.0, 400.0)	400.0 (0.0, 800.0)	0.091
Plasma	0.0 (0.0, 400.0)	0.0 (0.0, 400.0)	200.0 (0.0, 400.0)	0.057
Platelet	0.0 (0.0, 0.0)	0.0 (0.0, 10.0)	0.0 (0.0, 10.0)	0.11
Cryoprecipitate	0.0 (0.0, 10.0)	0.0 (0.0, 10.0)	10.0 (0.0, 10.0)	0.003
Intraoperative blood loss(ml), Median (IQR)	300.0 (200.0, 500.0)	300.0 (200.0, 500.0)	300.0 (200.0, 500.0)	0.262
Intraoperative urine output(ml), Median (IQR)	1000.0 (800.0, 1500.0)	1100.0 (800.0, 1800.0)	1200.0 (700.0, 2000.0)	0.28
Duration of surgery(min), Median (IQR)	260.0 (215.0, 315.0)	277.5 (226.2, 367.0)	300.0 (240.0, 375.0)	0.029
Duration of anesthesia(min), Median (IQR)	300.0 (255.0, 355.0)	327.5 (272.0, 404.5)	340.0 (270.0, 415.0)	0.087
DHCA, n (%)	2 (1.2)	0 (0)	2 (6.2)	0.116
Nasopharyngeal temperature(℃), Median (IQR)	32.4 (31.8, 32.9)	31.9 (31.5, 32.4)	32.0 (27.4, 32.5)	0.023
Anal temperature(℃), Median (IQR)	33.1 (32.4, 33.5)	32.7 (32.0, 33.1)	32.7 (28.5, 33.2)	0.016
Minimum intraoperative Hb level(g/L), Median (IQR)	6.8 (5.8, 8.2)	7.2 (6.1, 8.4)	6.8 (6.2, 7.5)	0.695
Minimum intraoperative Hct level(%), Median (IQR)	21.2 (19.0, 25.0)	23.0 (18.0, 26.2)	21.0 (19.0, 24.0)	0.584
Minimum intraoperative PaO_2(mmHg), Median (IQR)	260.0 (152.0, 308.5)	235.0 (115.5, 269.0)	216.0 (145.5, 302.8)	0.28
Maximum intraoperative lactate level(mmol/L), Median (IQR)	3.5 (2.5, 5.2)	3.9 (2.2, 6.3)	4.6 (2.9, 6.2)	0.303

BMI, body mass index; ASA, American Society of Anesthesiologists; ECMO, extra-corporeal membrane oxygenation; IABP, intra-aortic balloon pump; EF, ejection fraction; LA, left atrial; RA, right atrial; LV, left ventricular; RV, right ventricular; TB, total bilirubin; DB, direct bilirubin; ALT, alanine aminotransferase; AST, aspartate aminotransferase; BUN, blood urea nitrogen; Cr, creatinine; WBC, white blood cell; Lym, lymphocyte; Neu, neutrophil; SII, systemic immune-inflammation index; NLR, neutrophil-to-lymphocyte ratio; PLR, platelet-to-lymphocyte ratio; MPV, mean platelet volume; RBC, red blood cell; RDW, red blood cell distribution width; INR, international normalized ratio; BNP, brain natriuretic peptide; CABG, coronary artery bypass graft surgery; DHCA, deep hypothermic circulatory arrest; Hb, hemoglobin; Hct, hematocrit; PaO2, partial pressure of oxygen in arterial blood.

Supplementary Table 8. Postoperative outcomes of the patients undergoing on-pump surgery excluding heart transplantation with AKI of different stages. AKI, acute kidney injury……

	AKI Stage 1 (n = 169)	AKI Stage 2 (n = 34)	AKI Stage 3 (n = 33)	*p value*
**Postoperative outcomes**				
Duration of mechanical ventilation in ICU(h), Median (IQR)	18.0 (8.0, 22.4)	21.3 (18.5, 59.0)	22.8 (17.0, 60.8)	< 0.001
>24h	33 (19.5)	17 (50)	16 (48.5)	< 0.001
>48h	10 (5.9)	10 (29.4)	12 (36.4)	< 0.001
Reintubation, n (%)	7 (4.2)	4 (11.8)	5 (15.2)	0.016
Tracheostomy, n (%)	4 (2.4)	3 (8.8)	6 (18.2)	0.002
Maximum postoperative PCT level(ng/ml), Median (IQR)	2.4 (0.7, 5.6)	6.0 (3.2, 10.9)	16.3 (6.0, 38.6)	< 0.001
Initiation of CRRT, n (%)	3 (1.8)	2 (5.9)	16 (48.5)	< 0.001
Cardiac arrest, n (%)	3 (1.8)	0 (0)	5 (15.2)	0.002
Redo surgery, n (%)	8 (4.8)	4 (12.1)	9 (27.3)	< 0.001
Postoperative ECMO/IABP/or both support, n (%)	8 (4.7)	9 (26.5)	14 (42.4)	< 0.001
LOS-ICU(d), Median (IQR)	4.0 (3.0, 6.0)	6.5 (4.0, 12.8)	10.0 (6.0, 21.0)	< 0.001
LOS (d), Median (IQR)	22.0 (18.0, 28.0)	25.0 (21.0, 32.8)	24.0 (18.0, 42.0)	0.127
Postoperative LOS (d), Median (IQR)	15.0 (12.0, 20.0)	20.0 (15.2, 26.0)	18.0 (14.0, 33.0)	0.004
In-hospital mortality, n (%)	9 (5.3)	6 (17.6)	13 (39.4)	< 0.001
30-day mortality, n (%)	7 (4.1)	6 (17.6)	10 (30.3)	< 0.001

ICU, intensive care unit; PCT = procalcitonin; CRRT, continuous renal replacement therapy; LOS = length of stay; LOS-ICU, length of stay in ICU.
