# Supplementary material for: The incidence, risk factors, and prognosis of acute kidney injury in patients after cardiac surgery
Source: Front Cardiovasc Med. 2024 Jul 16;11:1396889. doi: 10.3389/fcvm.2024.1396889 (PMC11286402; doi:10.3389/fcvm.2024.1396889)
Supplement: Supplementary file 1 [file Datasheet1.zip › Data Sheet 1_v1/Supplementary Table 9 and 10.pdf]

Supplementary Table 9. Univariate analysis of risk factors for AKI after cardiac surgery. AKI, acute kidney injury……

Predictors	Odds Ratio	95% confidence interval		*P* value
		Lower	Upper	
Age, per 5-yr increase	1.19	1.12	1.28	<0.001
ASA≥3	4.33	2.78	6.74	<0.001
Atrial fibrillation	2.55	1.71	3.8	<0.001
TB	1.03	1.01	1.05	0.002
DB	1.08	1.04	1.12	<0.001
Albumin	0.92	0.88	0.96	<0.001
Cr	1.02	1.01	1.03	<0.001
eGFR	0.98	0.98	0.99	<0.001
WBC	1.08	1.03	1.14	0.002
Lym	0.54	0.43	0.69	<0.001
Neu	1.13	1.07	1.19	<0.001
NLR	1.11	1.07	1.15	<0.001
PLR	1.00	1.00	1.00	0.04
SII	1.00	1.00	1.00	<0.001
RBC	0.77	0.62	0.96	0.023
Hb categories	0.69	0.56	0.85	<0.001
MPV	1.17	1.04	1.33	0.002
Platelet	1.00	0.99	1.00	0.001
RDW	1.26	1.13	1.4	<0.001
INR categories	3.00	2.18	4.13	<0.001
D-dimer	1.22	1.11	1.34	<0.001
BNP categories	1.93	1.56	2.38	<0.001
EF	0.98	0.97	1.00	0.013
LA volume	1.01	1.00	1.01	<0.001
RA volume	1.01	1.00	1.01	0.005
LV volume	1.00	1.00	1.01	0.01
Surgical types	1.10	1.03	1.18	0.004
Aortic dissection surgery	6.78	3.57	12.86	<0.001
Intraoperative crystalloid infusion	1.00	1.00	1.00	<0.001
Intraoperative erythrocyte transfusion volume	1.00	1.00	1.00	<0.001
Intraoperative plasma transfusion volume	1.00	1.00	1.00	<0.001
Intraoperative platelet transfusion volume	1.15	1.1	1.2	<0.001
Intraoperative cryoprecipitate transfusion volume	1.15	1.11	1.19	<0.001
Intraoperative total transfusion volume	1.07	1.05	1.09	<0.001
Intraoperative blood loss	1.00	1.00	1.00	0.004
Intraoperative urine output	1.00	1.00	1.00	0.004
Duration of surgery	1.01	1.01	1.01	<0.001
Duration of anesthesia	1.01	1.01	1.01	<0.001
Nasopharyngeal temperature	0.81	0.75	0.88	<0.001
Anal temperature	0.72	0.65	0.80	<0.001
Minimum intraoperative Hb level	0.91	0.83	1.00	0.042
Minimum intraoperative Hct level	0.95	0.92	0.98	0.004
Maximum intraoperative lactate level	1.48	1.35	1.61	<0.001

ASA, American Society of Anesthesiologists; eGFR=estimated glomerular filtration rate; EF, ejection fraction; LA, left atrial; RA, right atrial; LV, left ventricular; RV, right ventricular; TB, total bilirubin; DB, direct bilirubin; Cr, creatinine; WBC, white blood cell; Lym, lymphocyte; Neu, neutrophil; SII, systemic immune-inflammation index; NLR, neutrophil-to-lymphocyte ratio; PLR, platelet-to-lymphocyte ratio; MPV, mean platelet volume; RBC, red blood cell; RDW, red blood cell distribution width; INR, international normalized ratio; BNP, brain natriuretic peptide; Hb, hemoglobin; Hct, hematocrit.

Supplementary Table 10. Multivariate analysis of risk factors for AKI after cardiac surgery. AKI, acute kidney injury……

Predictors	Odds Ratio	95% confidence interval		*P* value
		Lower	Upper	
Age, per 5-yr increase	1.218	1.105	1.344	<0.001
Atrial fibrillation	2.133	1.219	3.733	0.008
ASA≥3	3.528	1.972	6.313	<0.001
MPV	1.220	1.026	1.451	0.024
BNP <100 *(reference)*	1	1	1	0.07
BNP 100-400	1.720	1.024	2.889	0.040
BNP >400	2.601	1.421	4.759	0.002
Maximum intraoperative lactate level	1.423	1.258	1.609	<0.001
Intraoperative cryoprecipitate transfusion volume	1.445	1.265	1.652	<0.001

ASA, American Society of Anesthesiologists; MPV, mean platelet volume; BNP, brain natriuretic peptide.
