# Supplementary figures and images for: The incidence, risk factors, and prognosis of acute kidney injury in patients after cardiac surgery
Source: Front Cardiovasc Med. 2024 Jul 16;11:1396889. doi: 10.3389/fcvm.2024.1396889 (PMC11286402; doi:10.3389/fcvm.2024.1396889)

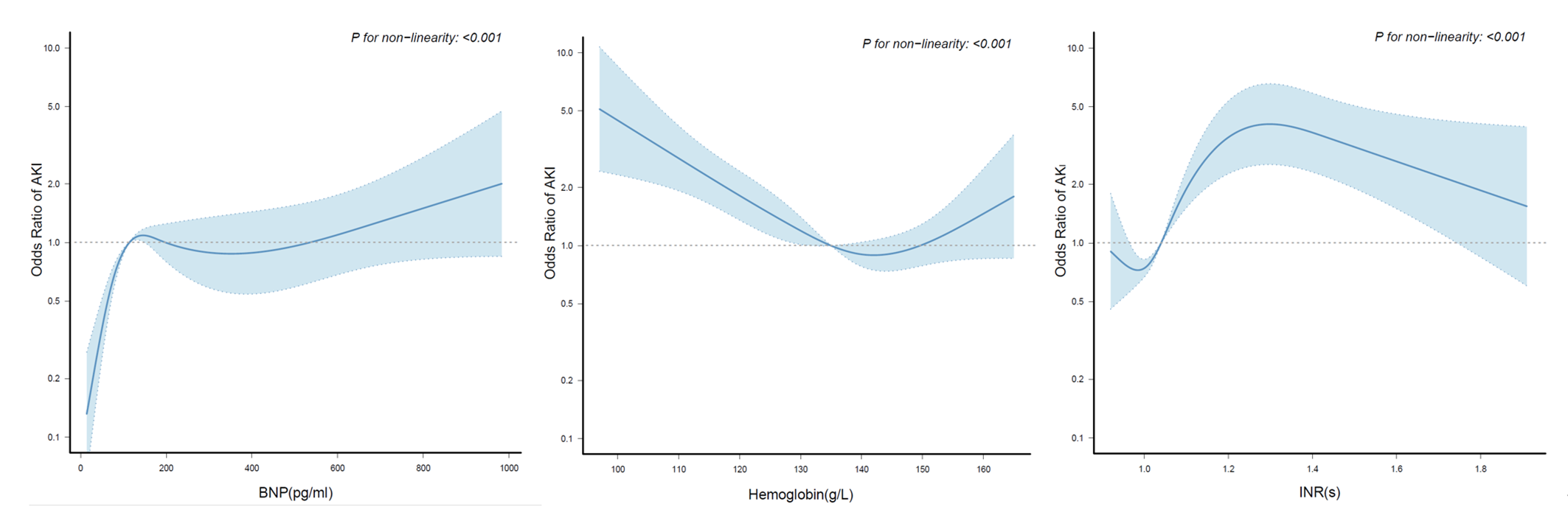

Supplement: Supplementary Figure 1 — Non-linear associations of BNP, INR, and Hb with AKI. [file Image1.tif]

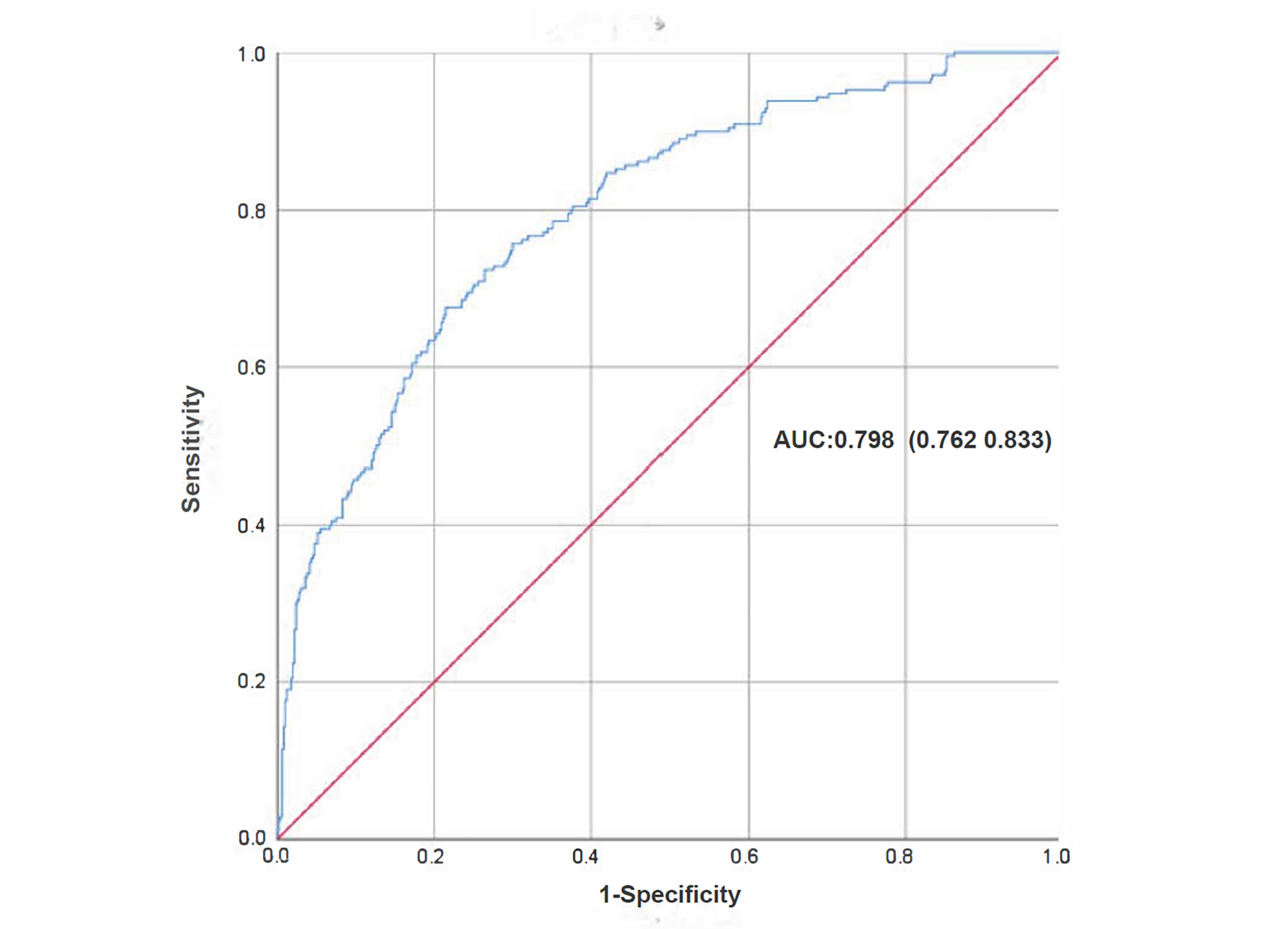

Supplement: Supplementary Figure 2 — Sensitivity analysis: Receiver-operating characteristic (ROC) curve for evaluating the discrimination performance of the logistic model in the study cohort. [file Image2.tif]
